# Merging FT-IR and NGS for simultaneous phenotypic and genotypic identification of pathogenic *Candida* species

**DOI:** 10.1371/journal.pone.0188104

**Published:** 2017-12-04

**Authors:** Claudia Colabella, Laura Corte, Luca Roscini, Volha Shapaval, Achim Kohler, Valeria Tafintseva, Carlo Tascini, Gianluigi Cardinali

**Affiliations:** 1 Department of Pharmaceutical Sciences—Microbiology, University of Perugia, Perugia (Italy); 2 Department of Mathematical Sciences and Technology, Norwegian University of Life Sciences, Norway; 3 Azienda Ospedaliera dei Colli—Ospedale Cotugno, Napoli, Italy; 4 CEMIN, Centre of Excellence on Nanostructured Innovative Materials—Department of Chemistry, Biology and Biotechnology—University of Perugia, Perugia, Italy; Institute of Microbiology, SWITZERLAND

## Abstract

The rapid and accurate identification of pathogen yeast species is crucial for clinical diagnosis due to the high level of mortality and morbidity induced, even after antifungal therapy. For this purpose, new rapid, high-throughput and reliable identification methods are required. In this work we described a combined approach based on two high-throughput techniques in order to improve the identification of pathogenic yeast strains. Next Generation Sequencing (NGS) of ITS and D1/D2 LSU marker regions together with FTIR spectroscopy were applied to identify 256 strains belonging to *Candida* genus isolated in nosocomial environments. Multivariate data analysis (MVA) was carried out on NGS and FT-IR data-sets, separately. Strains of *Candida albicans*, *C*. *parapsilosis*, *C*. *glabrata* and *C*. *tropicalis*, were identified with high-throughput NGS sequencing of ITS and LSU markers and then with FTIR. Inter- and intra-species variability was investigated by consensus principal component analysis (CPCA) which combines high-dimensional data of the two complementary analytical approaches in concatenated PCA blocks normalized to the same weight. The total percentage of correct identification reached around 97.4% for *C*. *albicans* and 74% for *C*. *parapsilosis* while the other two species showed lower identification rates. Results suggested that the identification success increases with the increasing number of strains actually used in the PLS analysis. The absence of reliable FT-IR libraries in the current scenario is the major limitation in FTIR-based identification of strains, although this metabolomics fingerprint represents a valid and affordable aid to rapid and high-throughput to clinical diagnosis. According to our data, FT-IR libraries should include some tens of certified strains per species, possibly over 50, deriving from diverse sources and collected over an extensive time period. This implies a multidisciplinary effort of specialists working in strain isolation and maintenance, molecular taxonomy, FT-IR technique and chemo-metrics, data management and data basing.

## Introduction

The correct identification and classification of fungi is essential for basic biological research such as the assessment of biodiversity, conservation, taxonomy and evolutionary biology and for those applications in which humanity and biodiversity intersect (agriculture, ecology, bioremediation and pathology) [1, 2].

To understand the biodiversity, the ecological roles and the geographical distribution of pathogenic fungi, DNA barcoding was proven to be a powerful tool with enormous potential [3]. DNA barcoding is a global initiative designed to provide rapid, accurate, and automatable species identifications by using short, standardized gene regions as internal species markers [4]. The critical issue underlying barcoding is accuracy, defined in taxonomic terms as the capability of unbiased and unequivocal identification at the species level. Accuracy depends especially on the extent of, and the separation between, intra-specific variation and inter-specific divergence in the selected marker creating a significant barcoding ‘‘gap” between intra- and inter-specific variation [5]. The sequences that are unique for a single species make identification easier, but their lack of universality hamper their amplification and therefore the whole procedure [3, 6].

Many barcode markers have been described for fungi [7–17]. For yeasts, the D1/D2 domain of the nuclear large ribosomal subunit (LSU) was adopted for characterizing species long before the concept of DNA barcoding was promoted [8, 18, 19]. Among the region of the ribosomal operon, the internal transcribed spacer (ITS) showed a relatively good level of identification, displaying the most clearly defined barcoding gap between intra and interspecific variation for most species of fungi and has been adopted as their universal standard barcoding region [17]. In addition, ITS displays high robust PCR amplification fidelity (> 90% success rate), a Probability of Correct Identification (PCI) of about 70% and pertinence to a broad range of sample conditions [13]. The rDNA operon consist of multiple copies ranging from around 50 to 100 per haploid genome in fungi [20, 21].

Different processes can occur within individual sequence heterogeneity in the ribosomal repeat. In some cases, these can complicate the analysis using ITS sequencing, such as intra- and inter-taxon hybridization with the loss of the homogenization of the ribosomal repeat in a broad range of species. In order to increase the accuracy of species identification robust primers for secondary barcodes were explored [13, 22].

Alongside with the development of genetic techniques, phenotyping techniques also undergo enormous development. Currently, there is a few modern phenotyping techniques that based on their robustness and sensitivity could be considered as Next Generation Phenotyping (NGP) techniques. One of them is FT-IR spectroscopy, which is an emerging technique to characterize and identify fungi in many different fields like food microbiology, medical diagnostics and microbial ecology [23–26]. The method has been successfully applied for the identification of fungal genera such as *Penicillum* and *Fusarium* spp [24], fungal phyto-pathogenes [23], for the differentiation of *Aspergillus* and *Penicillium* at species and strain level [25], yeast food-related strains [27, 28] and for pathogenic strains belonging to the *Candida* genus [29–33]. FT-IR spectroscopy represents thus a multi-molecular method to apply in a clinical setting alone or in combination with other analytical techniques.

FT-IR spectroscopy was established for microbial identification by Naumann and co- workers in the 90ies [34]. The basic principle of FT-IR absorbance spectroscopy is the absorption of vibrating chemical bonds in sample molecules at specific frequencies represented by the infrared spectrum. FT-IR spectroscopy is a high-throughput technique which does not require extended sample manipulation and allowing to achieve massive and rapid molecular information of samples at very low running costs [30, 34–36].

Recent advances in the development of high-throughput sample preparation techniques, allow cultivation of fungi in 96-microwell plates and measurement by high-throughput FT-IR spectroscopy employing 384-well plates for FT-IR measurements after one day growth for yeasts and five days growth for filamentous fungi [37–39]. Since the FT-IR phenotype represents a biochemical fingerprint of the cells, growth media and growth conditions need to be strictly controlled [38, 40–42]. In contrast, the high sensitivity of the FT-IR biochemical fingerprint towards phenotypic changes offers a great opportunity to elucidate taxonomy by acquiring FT-IR spectra of microorganisms using different, but defined and tailored growth media [38], an approach which is used for genome-wide phenotyping via growth parameters [43].

Identification of microorganisms via FT-IR fingerprints can be accomplished by the use of spectral databases. Comprehensive databases have been established covering a large range of species and genera by the use of reference strains [44]. When suitable databases are established, spectra of unknown strains can be compared with the reference database spectra and strains can be rapidly identified at genus, species and sometimes even at the strain level. Since identification of pathogenic yeast is crucial for mortality rates of hospitalised patients [32], the implementation of rapid identification via FT-IR spectroscopy may reduce death rates in patients and social costs related infectious diseases [45, 46].

The aim of this paper is to compare identification and classification of pathogenic *Candida* yeasts species via FT-IR spectroscopy and DNA barcoding as well as to develop more accurate and rapid approaches to identify pathogens. To this purpose, a large set of pathogenic yeasts was employed including the three species, *C*. *albicans*, *C*. *glabrata* and *C*. *tropicalis*, which are commonly found in medical environments. In addition, *C*. *parapsilosis* strains that are commonly found in natural and food environments [47] were analysed by FT-IR spectroscopy and DNA barcoding.

In order to evaluate the capability of FT-IR spectroscopy and DNA barcoding in describing inter- species and intra-species variability, results of both methods were integrated into one data model by so-called consensus principal component analysis (CPCA). In order to account for the problems deriving from the inherent species variability, a panel of several strains per species was used. We further evaluated to what extend FT-IR spectroscopy and DNA barcoding can be used for identification. In order to accomplish this, we performed identification based on FT-IR spectroscopy via multivariate analysis and we considered taxonomic issues, such as the relation of strains to the type strain.

## Materials and methods

### Strains and growth conditions

We analysed a collection of 286 strains, belonging to opportunistic species of *Candida* genus isolated from two Italian Hospitals (Pisa and Udine) and included in the Cemin Microbial Collection of the Microbial Genetics and Phylogenesis Laboratory of Cemin (Centre of Excellence on Nanostructured Innovative Materials for Chemicals, Physical and Biomedical Applications—University of Perugia). All strains were isolated from patient blood cultures and extensively described in a medical ecology paper [48].

Twelve species were isolated from both hospitals, with *C*. *albicans*, *C*. *glabrata*, *C*. *parapsilosis* and *C*. *tropicalis*, representing the vast majority of the isolates. 256 strains of these four species and the respective type strains were employed in this study (S1 Table).

All strains were stored at -80°C in 17% glycerol right after isolation. Cultivation was carried out on YEPDA (YEPD with 1.7% agar) at 37°C, following the current procedures. To generate cell biomass needed for the analysis, the strains were grown in YEPD broth (Yeast extract 1%, Peptone 1%, Dextrose 1%; all chemicals from Biolife, Italy - http://www.biolifeitaliana.it/) at 37°C with 150 rpm shaking.

### Molecular analysis and bioinformatics tools

Genomic DNA was extracted as indicated by Cardinali et al [49] ITS1, 5.8S, ITS2 rDNA genes and D1/D2 domain of the LSU were amplified with FIREPole^®^ Taq DNA Polymerase (Solis BioDyne, Estonia), using ITS1 (5’-TCCGTAGGTGAACCTGCGG)—NL4 (GGTCCGTGTTTCAAGACGG) primers. The amplification protocol was carried out as follows: initial denaturation at 94°C for 3 min, 30 amplification cycles (94°C for 1 min, 54°C for 1 min and 72°C for 1 min) and final extension at 72°C for 5 min. Amplicons were subjected to electrophoresis on 1.5% agarose gel (Gellyphor, EuroClone, Italy). Amplicons were sequenced with NGS PlexWell^™^ technologies (http://www.seqwell.com/) with the same primers used for the generation of the amplicons. The reads of each strain, contained in FASTQ file, were analysed with Geneious R9 (v. 9.1.5, Biomatters, Auckland, New Zealand - http://www.geneious.com/). Identification was carried out according to the criteria indicated in the taxonomic papers dealing with LSU [8] and ITS [17, 22, 50, 51].

In order to obtain distance matrices for the four major species, all the consensus sequences were aligned with the corresponding type strain using pairwise alignment in Geneious software (Biomatters, New Zeland) (S2 and S3 Tables). The distance matrices were calculated through the base of percentage of identical bases/residues and exported as tsv files in Microsoft Excel ^®^.

### FT-IR measurements

For FT-IR analysis, the selected strains were grown over night in YNB (added with 2% dextrose, 1.7% agar—all products from Biolife). For each sample one colony was transferred with a calibrated platinum loop from the plate to Eppendorf tubes containing 200μl of pure water (HPLC Gradient Grade—J.T. Baker - http://www.jtbaker.com/). Of each suspension, 35μl were transferred to an IR-light-transparent silicon 96-well microtiter plate (Bruker, Germany). The samples were dried at 42°C to form films of uniform thickness in order to minimize the interference of scattering effects during the acquisition of FT-IR absorbance spectra.

FT-IR measurements were performed in transmission mode. For each sample three technical replicates were performed. All spectra were recorded in the range between 4000 and 400 cm^-1^ with a TENSOR 27 FT-IR spectrometer, equipped with HTS-XT accessory for rapid automation of the analysis (Bruker, Germany). Spectral resolution was set at 4 cm^-1^, sampling 128 scans per sample. The OPUS version 6.5 software (Bruker, Germany) was used to carry out the quality test and to obtain a matrix of raw spectra, which was subsequently exported as ASCII file.

### Pre-processing of FT-IR data

The measured FT-IR raw data consisted of 780 spectra including technical replicates. The data set was reduced to 260 spectra by averaging over technical replicates. Whole raw spectra were pre-processed with the second derivative function by the Savitzky-Golay algorithm and 15 smoothing points [52]. The 3050–2800 cm^-1^ and 1800–700 cm^-1^ intervals were considered.

Then, Extended Multiplicative Signal Correction (EMSC) taking into account linear and quadratic components was applied [53]. Pre-processing by second derivative and EMSC is done, in order to remove physical variations such as baseline variations and variations due to the thickness of the film of microbial cells used for FT-IR transmission spectroscopy [54].

### Multivariate analysis of FT-IR and NGS data

#### Consensus principal component analysis

**Fig 1:**
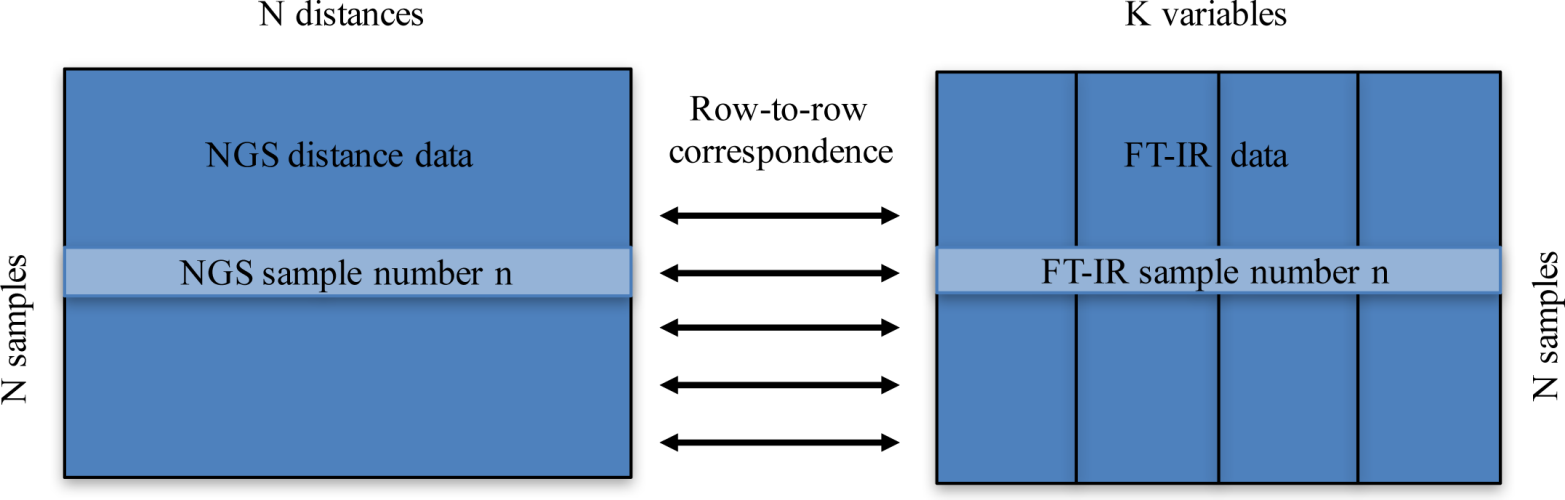
For CPCA of FT-IR and NGS data a row-to-row correspondence needs to be obtained. In order to integrate NGS data and FT-IR data in one data model, consensus principal component analysis is applied (CPCA). For CPCA, the data is organized such that a row-to-row correspondence between the different data blocks is obtained. The NGS distance data matrix contains N samples as rows and as columns the variables, which are the distances to all samples. The FT-IR data contains N samples as rows and as columns the absorbance values at different wavenumbers. The FT-IR data is further split into four data blocks according to groups of chemicals.

Consensus principal component analysis (CPCA) was applied in order to integrate NGS distance data and FT-IR absorbance data in one data model. CPCA is a so-called multiblock method that can be used to connect different types of data [55]. For CPCA, the data was organized such that a row-to-row correspondence was obtained for NGS distance data and FT-IR absorbance data (Fig 1).

The FT-IR data was further split into four blocks, namely the FT-IR absorbance values from 3050–2800 cm^-1^ were defined as block one, the region from 1800–1500 cm^-1^ as block two, the region from 1500–1200 cm^-1^ as block three and the region from 1200–700 cm^-1^ as block four. The NGS distance matrix was defined as block five. Each block was normalized to unit variance in order to set all blocks on the same footing. This is done by normalizing each block by the Frobenius norm, thus achieving the same weight for each block [56]. CPCA is based on principal component analysis. CPCA is equivalent to performing a PCA on all five concatenated blocks, where all blocks are normalized by the Frobenius norm. By CPCA, global scores and block scores are calculated. The global scores are equivalent to PCA scores obtained on concatenated and normalized blocks. They represent the consensus of all blocks and allow studying global sample and variable variation patterns. In addition to global scores, CPCA calculates block parameters, so-called block scores and block loadings.

The block scores can be used to study the block sample variation patterns for each consensus component, i.e. the sample variation in each block that contributes to the consensus. How strongly every block contributes to the consensus can be estimated by explained block variances, which are calculated for each block. In order to study correlations between variables between and within the blocks, correlation loading blocks can be used [57]. In correlation loading plots, the correlations between the global scores and the FT-IR and genetic distances matrices are plotted. In addition, correlations between global scores and group variables for each species are visualized in the same plots. Species groups are represented by so-called indicator variables. Each species is represented by one column of indicator variables, where a strain obtains the value one if it belongs to a species and zero otherwise.

For the correlation loading plots, we multiply the genetic distance variables and the FT-IR second derivative data by minus one in order to facilitate the interpretation of the correlations with the group indicator variables. In second derivative spectra, bands appear as negative peaks and are thus inversely correlated to concentrations of chemical compounds. The multiplication by minus one turns the negative correlation into a positive correlation, which facilitates interpretation. A similar argument applies for the genetic distance matrices.

#### Identification by Partial Least Squares Discriminant Analysis

The rationale of Partial Least Squares Regression (PLSR) [58] was to use this system to select FT-IR wavelengths to establish classification models able to cluster strains in species according to the ITS clustering. In this context, ITS classification acts as taxonomic reference being the gold standard of fungal barcoding [17]. In order to establish models, the data matrix X of FT-IR spectra is regressed on a matrix Y of indicator variables containing group labels. When PLSR is used together with a matrix of group indicator variable a matrix Y, it is called Partial Least Squares Discriminant Analysis (PLSDA) and widely used for the identification of microorganisms.

The ***optimization of the model*** was done via cross-validation. A leave-one-out cross-validation (CV) procedure was used, where one strain was taken out at a time and used for validation. The four type strains were always included in the calibration model and never used for validation. Therefore, the CV contained 260 segments. The optimal number of principal components (AOpt) was determined as the one, which did not yield significantly higher MCR than the model with the minimum MCR. The MCR was calculated as a fraction of the misclassified samples by the total number of samples. The statistical significance was evaluated by a binomial test.

To ***validate the established model***, a cross-model-validation (CMV) was done [59]. A leave-one-out CMV was performed in the following manner. In each step of CMV one strain was left aside and a leave-one-out CV model was established on the rest of the samples as described above. Thus, the left-out strain was not included in the calibration model and identification was performed on the basis of similarity to other strains belonging to the same species. The sample, which is left aside, was used for validation and the misclassification rate was stored. As for the CV, type strains were not taken out and used for validation. Thus, the CMV consisted of 260 segments, where at each segment a 259-fold CV was done. The final CMV error is the mean of all the errors. The CMV error allows the control of stability and reliability of established classification models. A stable model is expected to show a CMV error, which is comparable to the CV error of the model.

#### Correlation analysis between NGS and FT-IR distance matrices

In order to correlate the two different data-sets, Mantel test analysis were carried out. Mantel's test is an approach that overcomes some of the problems inherent in explaining species-environment relationships. Mantel's test is a regression in which the variables are themselves distance or dissimilarity matrices summarizing pairwise similarities among sample locations. One advantage of Mantel's test is that, because it proceeds from a distance (dissimilarity) matrix, it can be applied to variables of different logical type (categorical, rank, or interval-scale data) [60]. In general, a Mantel test measures the correlation between two matrices typically containing measure of distance.

Correlation were performed using R environment software (http://www.R-project.org/) ade4 library, mantel.rtest and cor.test function for the estimation of the *p*-value with 9999 permutations using distance matrix calculated on the basis of the ITS and LSU markers and for the FT-IR matrices before and after the PLS model.

#### Identification by distances to type strains and central strains

Each strain was originally attributed to one of the four species by means of rapid clinical identification (CHROMagar) followed by MALDI-TOF, sequencing of the ITS and LSU D1/D2 regions and FT-IR spectroscopy. For molecular data, distance matrices were calculated through the base of percentage of identical bases/residues. For FT-IR data, distance matrixes were obtained on the basis of PLS loadings. After the PLS modeling as described above, a squared distance matrix was obtained with all the distances among objects in the PLS hyperspace.

For both sequencing and FT-IR data, four distance matrices, referred to as species matrices, were obtained. The species matrices contain the distances among members of the strains of each of the four species. As described elsewhere [61], the central strain (CS) of each species distribution was identified as that with the minimum sum of distances from all other strains. Type strains and central strains were named jointly as “reference strains”. The distances between each studied strain and: ***i*.** the four type strains (DTS); ***ii*.** the four central strains (DCS) and ***iii*.** the eight reference strains (DRS) were calculated.

Two identification approaches were tested:

1. **Single match approach**. The correct species attribution requires that the DTS or DCS be the lowest among the eight DRS values of each stain.
2. **Double match approach**. For each strain two identifications are carried, one with the TS and one with the CS. In both cases, the correct species attribution requires that the DTS or DCS is the lowest among respectively the four DTS and DCS values of each stain.

This can be summarized by the following logical expression.

*If* D (Si-TSj) < D (Si-TS) *⇒* Match = 1

D (Si-TSj) ≥ D (Si-TS) *⇒* Match = 0

## Results

### Connecting FT-IR and NGS data by consensus principal component analysis and grouping patterns in FT-IR data

In order to connect FT-IR data and DNA sequencing data in one data model, we applied consensus principal component analysis (CPCA) [55]. To this purpose, the FT-IR data was split into four blocks: The region from 3050–2800 cm^-1^ was defined as block one, the region from 1800–1500 cm^-1^ as block two, the region from 1500–1200 cm^-1^ as block three and the region from 1200–700 cm^-1^ as block four. The sequencing relationship matrix was defined as block five. In order to investigate global and block grouping patterns, block and global score plots are used [57].

**Fig 2:**
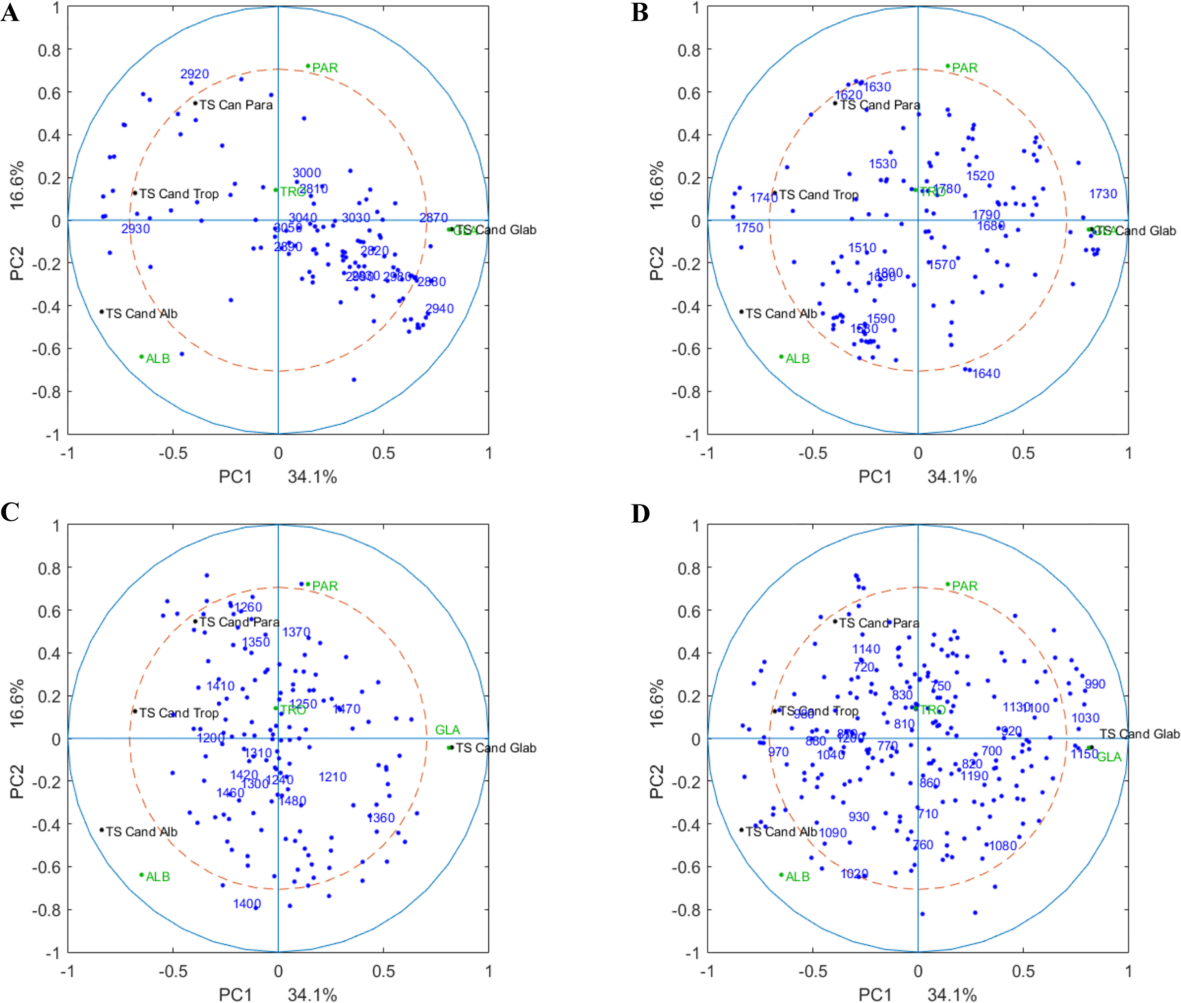
Correlation loading plot (PC1 and PC2) of NGS and FT-IR (block 1, 2, 3 and 4) with global scores. The correlation loading plots showing the correlation between the global scores of the CPCA analysis with the four different FT-IR blocks and the distance matrix of the genetic data. In addition, the correlations between the global scores and the genetic distance matrix and the indicator variables for each species are visualized in each plot. (A) Correlations between global scores and the lipid region (block 1, 3050–2800 cm^-1^), the genetic distance matrix and the species indicator variables; (B) correlations between the global scores and the mixed lipid and protein region (block 2, 1800–1500 cm^-1^), the genetic distance matrix and the species indicator variables; (C) correlations between the global scores and the mixed lipid, protein and polysaccharide region (1500–1200 cm^-1^), the genetic distance matrix and the species indicator variables and (D) correlations between the global scores and the polysaccharide region (1200–700 cm^-1^), the genetic distance matrix and the species indicator variables. Blue dots represent FT-IR wavelengths; black dots distances to TS Cand alb, TS Cand para, TS Cand glab and TS Cand trop represent type strains (TS) of the four species and green dots namely ALB, PAR, GLA and TRO represent the group variables (indicator variables).

The results showed that higher similarity between *C*. *albicans* and *C*. *tropicalis* was indicated in FT-IR data than NGS distance, whereas *C*. *tropicalis* seems to be more closely related to *C*. *parapsilosis*. These results are presented by the correlation loading plot (Fig 2D), where it could be seen that wavenumbers from different spectral regions are responsible for the separation of the *C*. *glabrata* from other three species, whereas *C*. *albicans*, *C*. *parapsilosis* and *C*. *tropicalis* are better separated by the linear sequence of variables within the 1200–700 cm^-1^ polysaccharide region.

**Fig 3:**
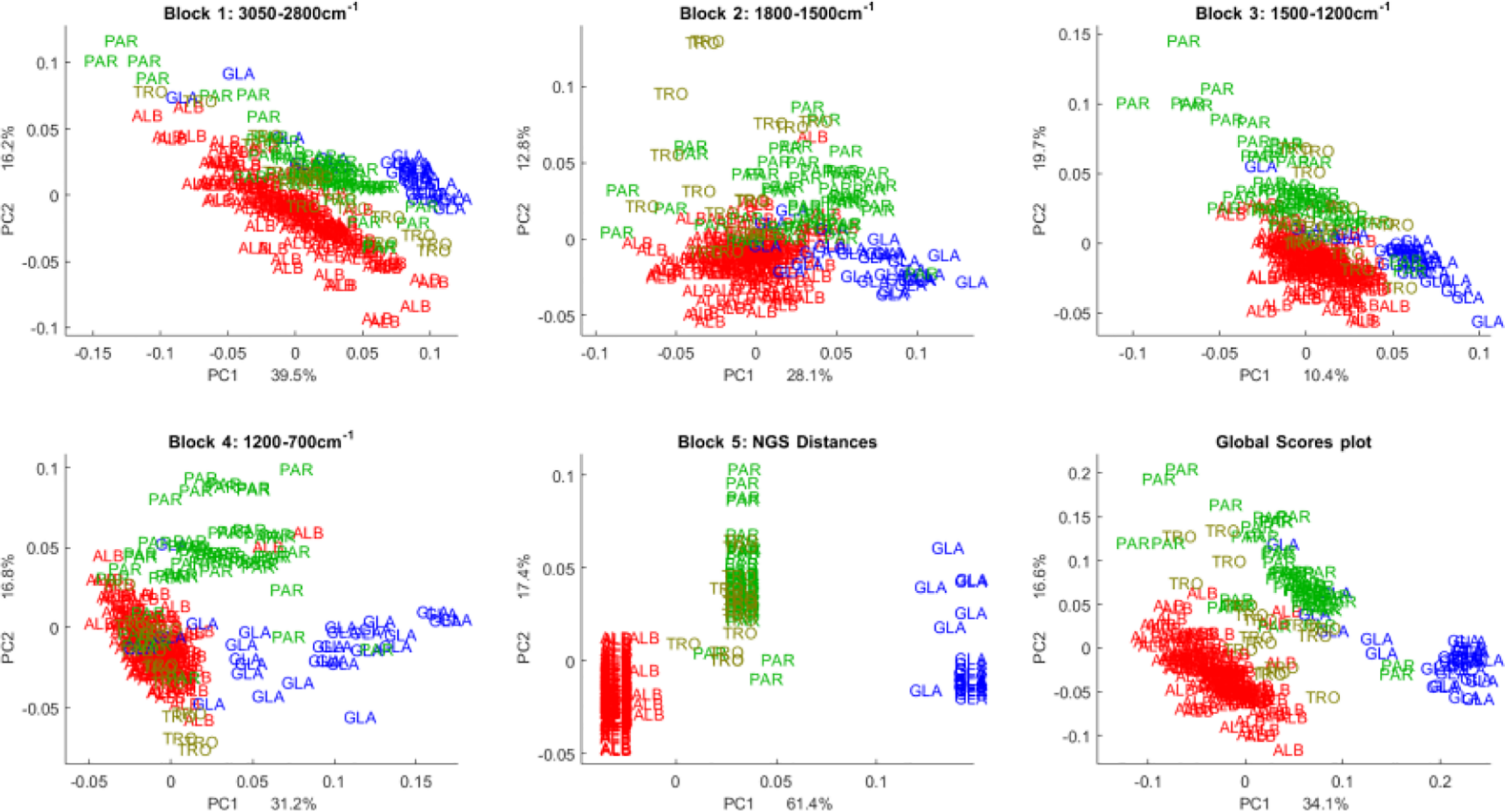
Score plots of CPCA (PC1 and PC2) analysis of genetic-NGS and phenotypic-FT-IR spectroscopic data of strains from four *Candida* species—*C*. *albicans*, *C*. *parapsilosis*, *C*. *glabrata* and *C*. *tropicalis*. The score plots of blocks 1–4 of CPCA analysis of FT-IR spectroscopy data, where block 1 is for lipid region (3050–2800 cm^-1^), block 2 is for mixed lipid and protein region (1800–1500 cm^-1^), block 3 is for mixed lipid, protein and polysaccharide region (1500–1200 cm^-^1) and block 4 is for polysaccharide region (1200–700 cm^-1^). The score plot of block 5 is for NGS data. The score plot of block 6 represents the global score plot of CPCA components one and two indicating the consensus of all blocks.

**Fig 4:**
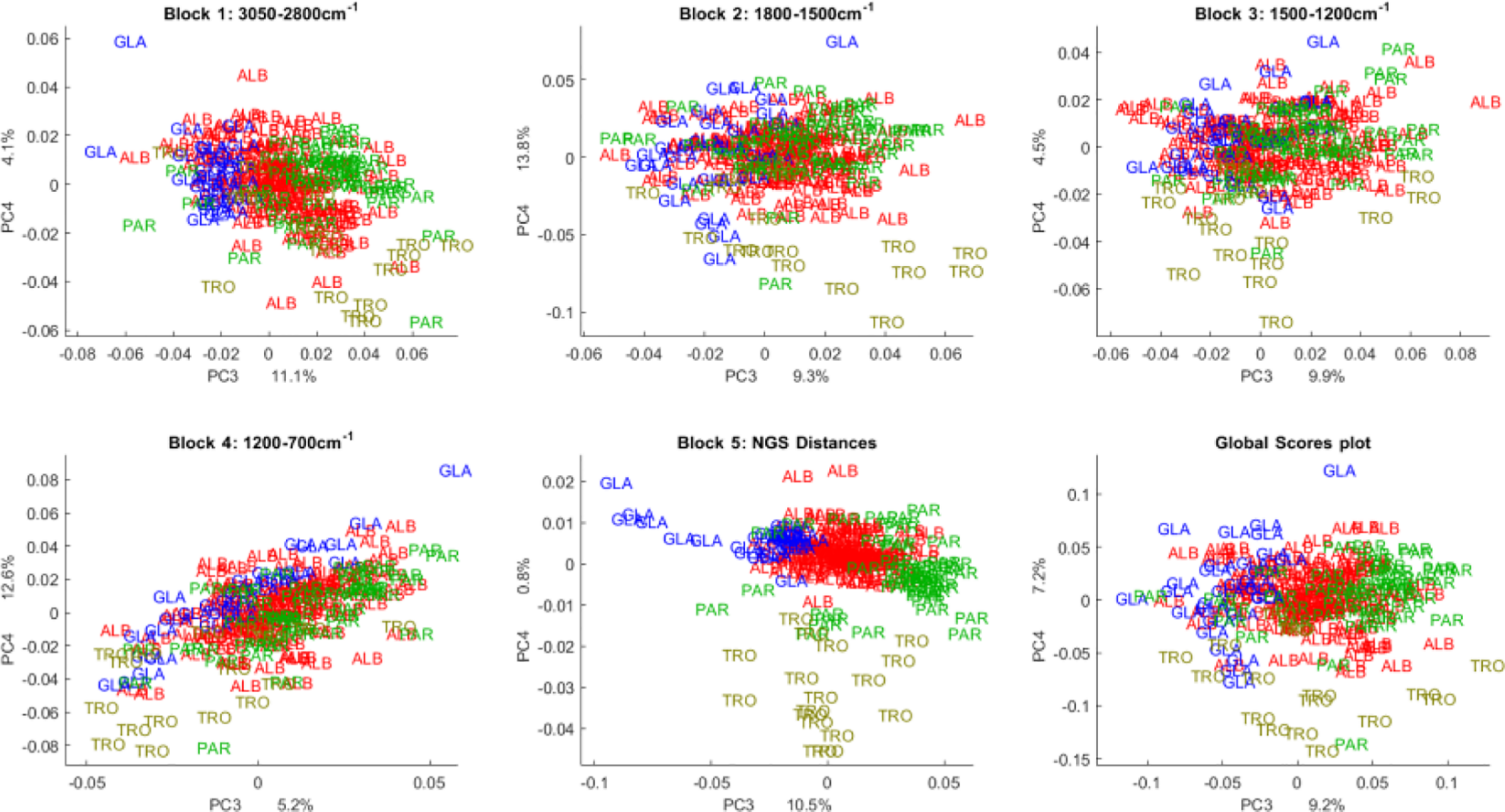
Score plots of CPCA (PC3 and PC4) analysis of genetic-NGS and phenotypic-FT-IR spectroscopic data of strains from four *Candida* species—*C*. *albicans*, *C*. *parapsilosis*, *C*. *glabrata* and *C*. *tropicalis*. The score plots of block 1–4 are for FT-IR spectroscopy data, where block 1 refers to the lipid region (3050–2800 cm^-1^), block 2 to the mixed lipid and protein region (1800–1500 cm^-1^), block 3 to the mixed lipid, protein and polysaccharide region (1500–1200 cm^-1^) and block 4 to the polysaccharide region (1200–700 cm^-1^). The score plot of block 5 refers NGS data. The score plot of block 6 represents the global score plot of CPCA components three and four indicating the consensus of all blocks.

CPCA of FT-IR data indicated that PC1 and PC2 in the first three data blocks separated *C*. *albicans–*ALB, *C*. *glabrata*–GLA, *C*. *parapsilosis*–PAR whereas *C*. *tropicalis*–TRO required additional PC3 and PC4 within the fourth data block to be clearly distinguished (Figs 3 and 4). Moreover, the first four PCs describe only 77.4% total variance, which required six additional components (up to PC10) to reach 92.9% value.

From the block score plots (Figs 3 and 4) it is obvious that intra-species variation captured by the FT-IR data (block one to four) is much larger than the NGS intra-species variation (block five). As shown previously, the phenotyping variability identified by FT-IR can be explained by a real chemical variability between the strains, and not by an instrumental variability, which is negligible [37]. Further, it was shown that the chemical variability between strains is mainly due to inherent chemical differences between strains if cultivation conditions are controlled strictly [37].

All block score plots for the first and second component (Fig 3) show a distinct separation of the three species—*C*. *albicans*, *C*. *parapsilosis*, *C*. *glabrata*—for both genetic and FT-IR data. It is interesting to note, that in the global score plot (block 6) the species *C*. *tropicalis* is separated and located between *C*. *albicans* and *C*. *parapsilosis* (Fig 3) while, in all block score plots for FT-IR and NGS data, *C*. *tropicalis* is overlapping with other species. A possible explanation is that whereas in block one, two, three and five, *C*. *tropicalis* is mixed with *C*. *parapsilosis*, block four shows an overlap of *C*. *parapsilosis* and *C*. *albicans*. Therefore, when combining the discriminant information contained in all four FT-IR blocks, a separation of all four species is to a large extent possible by only using the first two components of the FT-IR data. While *C*. *glabrata*, *C*. *albicans* and *C*. *parapsilosis* can be discerned by the first two components of all blocks, including lipid, protein and polysaccharide region, *C*. *tropicalis* is overlapping with other species for all blocks including the NGS data.

For all blocks, except the polysaccharide region, *C*. *tropicalis* is overlapping with *C*. *parapsilosis* for the first two components, while in the polysaccharide region *C*. *tropicalis* is overlapping with *C*. *albicans*. This can suggest that *C*. *glabrata*, *C*. *albicans* and *C*. *parapsilosis* are phenotypically and biochemically very different, while *C*. *tropicalis* and *C*. *parapsilosis* appear phenotypically and biochemically very similar. The separation between *C*. *tropicalis* and *C*. *parapsilosis* supported by the results of CPCA within the polysaccharide region can suggest that major differences are in the cell wall, which associates the majority of the cellular polysaccharides. It is interesting to note that *C*. *albicans* and *C*. *tropicalis*, although separated in all other FT-IR blocks, are similar in their polysaccharide profile, which is revealed in the block score plot of the first two components of the region 1200–700 cm^-1^. Further, the score plots of principal component three and four show clear separation of the *C*. *tropicalis* species for both NGS and FT-IR data (Fig 4).

The global score plot of CPCA components one and two represents the consensus of all blocks involved. We can see that the first two components of the global scores representing the consensus of all blocks, separate all species of *Candida* namely *C*. *albicans*, *C*. *parapsilosis*, *C*. *glabrata* and *C*. *tropicalis* very well.

Further, comparing the corresponding block score plots for all FT-IR regions and the genetic-NGS, we observe that all block score plots show a similar tendency as the global scores, but there are also clear differences in grouping patterns and explained variances, i.e. the contributions of each block to the global pattern.

In the correlation loadings plot between the global scores of the FT-IR and the NGS distance matrix, the genetic distance matrix are nicely correlated with the group indicator variables (Fig 2). For instance, the first component showed significant difference between *C*. *glabrata* and the other three species. Furthermore, by the second component the difference between the other three species (*C*. *albicans*, *C*. *parapsilosis* and *C*. *tropicalis*) is explained. In Fig 2A the fatty acid region of FT-IR explains mainly the difference between *C*. *glabrata* and the other species. Considering the first component, the ester band (around 1750) explains very well the difference between *C*. *glabrata* and the other species (Fig 2B) while by the second component the protein bands explain the difference among the other three species. The mixed region (Fig 2C) showed differences between *C*. *parapsilosis*, *C*. *tropicalis* and *C*. *albicans* while the carbohydrates region (Fig 2D) explain difference among all the four species considered.

### Classification based on discriminant PLSR

A classification model was built by the PLSR method and optimized by cross-validation (CV). The established model contained six PLS components and a total success rate (SR) value of 94.2% was achieved.

**Fig 5:**
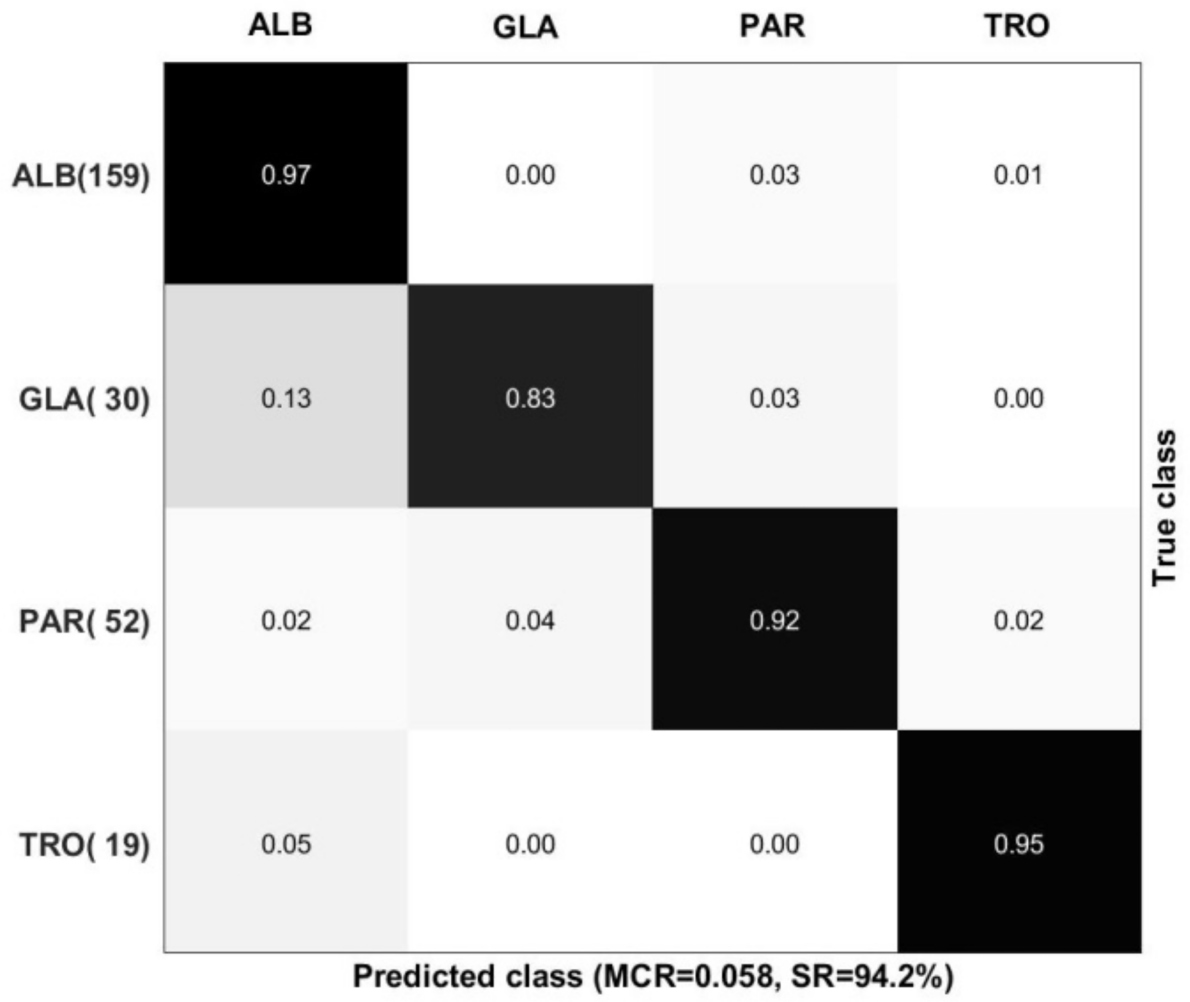
Confusion matrix for the cross-validated classification model. Errors are given as misclassification rate (MCR), which is the fraction of misclassified samples over the total number of samples. The success rate (SR) is given in percentage and equals SR = (1-MCR)*100. The number of samples in each group is specified in the left column with the true group affiliations. The predicted group is specified on the top of the matrix.

The corresponding confusion matrix is *C*. *glabrata* species with the SR equal to 83% (Fig 5).

The CMV was done in order to test the model performance and the error stability. The CMV error repeats exactly the CV error and the CMV success rate equals to 94.2%. This is an important property of the model suggesting that the cross-validated model is reliable and could perform well when used for prediction of a new strain.

It is important to put in mind that strains used for validation where not present in the dataset of strains used to establish the model. Both for CV and CMV validation was done by taking a strain completely out. Taking a strain completely out is the most stringent test that can be performed for validating the model and corresponds to the actual situation, where unknown strains need to be identified in hospitals or in source tracking in food industry.

### Correlation analysis between NGS and FT-IR distance matrices

In order to correlate the two different data-sets, Mantel test analysis were performed using distance matrix based on ITS and LSU markers and distances obtained with FT-IR in different conditions (Table 1).

**Table 1:** Mantel test analysis between NGS and FT-IR.

Conditions	Mantel *r*	*p* value
FT-IR whole spectrum	0.5725	0.0001
FT-IR block 1	0.2143	0.0001
FT-IR block 2	0.4729	0.0001
FT-IR block 3	0.3289	0.0001
FT-IR block 4	0.5465	0.0001
FT-IR block 1+2	0.4445	0.0001
FT-IR block 1+3	0.3002	0.0001
FT-IR block 1+4	0.5388	0.0001
FT-IR block 2+3	0.4761	0.0001
FT-IR block 2+4	0.5911	0.0001
FT-IR block 3+4	0.5485	0.0001
FT-IR—PLS PC 1	0.6456	0.0001
FT-IR—PLS PC 1–2	0.7062	0.0001
FT-IR—PLS PC 1–3	0.7121	0.0001
FT-IR—PLS PC 1–4	0.7089	0.0001
FT-IR—PLS PC 1–5	0.7071	0.0001
FT-IR—PLS PC 1–6	0.7090	0.0001
FT-IR—PLS PC 1–7	0.7075	0.0001
FT-IR—PLS PC 1–8	0.7072	0.0001
FT-IR—PLS PC 1–9	0.7018	0.0001
FT-IR—PLS PC 1–10	0.6955	0.0001

Mantel test data report the correlation between the distance matrix among strains calculated on the basis of LSU-ITS and the distance matrices among strains calculated with the FT-IR data in the conditions indicated in column 1. The *p* value reports the error probability of the corresponding mantel test. All Mantel analyses were carried out with 9999 permutations. FT-IR—PLS PC1 indicates that only the first principal component was used. Similarly, FT-IR—PLS PC 1:n indicates that all principal components from 1 to n were used to calculate the distance matrix.

Taxonomic analyses are carried out on the basis of the distance matrix among strains calculated on the basis of the sequences from well established molecular markers such as LSU and ITS. The idea of using the FT-IR technique as a sort of phenotypically proxy of the molecular markers relays on the possibility of applying cluster and factor analysis to select the right wavelengths for this use.

In order to test the quality of the FT-IR techniques compared to ITS and LSU sequencing, a series of Mantel tests were carried out between the distance matrix for all the strains obtained with the two molecular markers and the distance matrices obtained with different treatments of the FT-IR spectra. This test calculates the correlation (*r*) between distance matrices of the same size and gives also a *p* value on the quality of the correlation.

The whole FT-IR spectrum, with only basic pre-treatments, yielded 0.57 Mantel *r* that was higher than the values obtained with single blocks of IR region, and slightly lower than the combination of blocks 2 and 4 (*r* = 0.59) (Table 1). The distance matrix obtained with the first 10 principal components from the PLS analysis gave a 0.64 Mantel *r*. This analysis was repeated using the first principal component and then the combinations of consequent components from 1 to 10 (e.g PC1 and 2, PC 1 thru 3 etc).

The results of these tests showed that the best correlation with the LSU and ITS sequencing was obtained by using the first three principal components (*r* = 0.71). The last seven components contained a part of the overall spectral variability, but this was not correlated to the variations among species as detected by molecular markers.

### Distribution of the strains around the central and type strain: TS are not central

**Fig 6:**
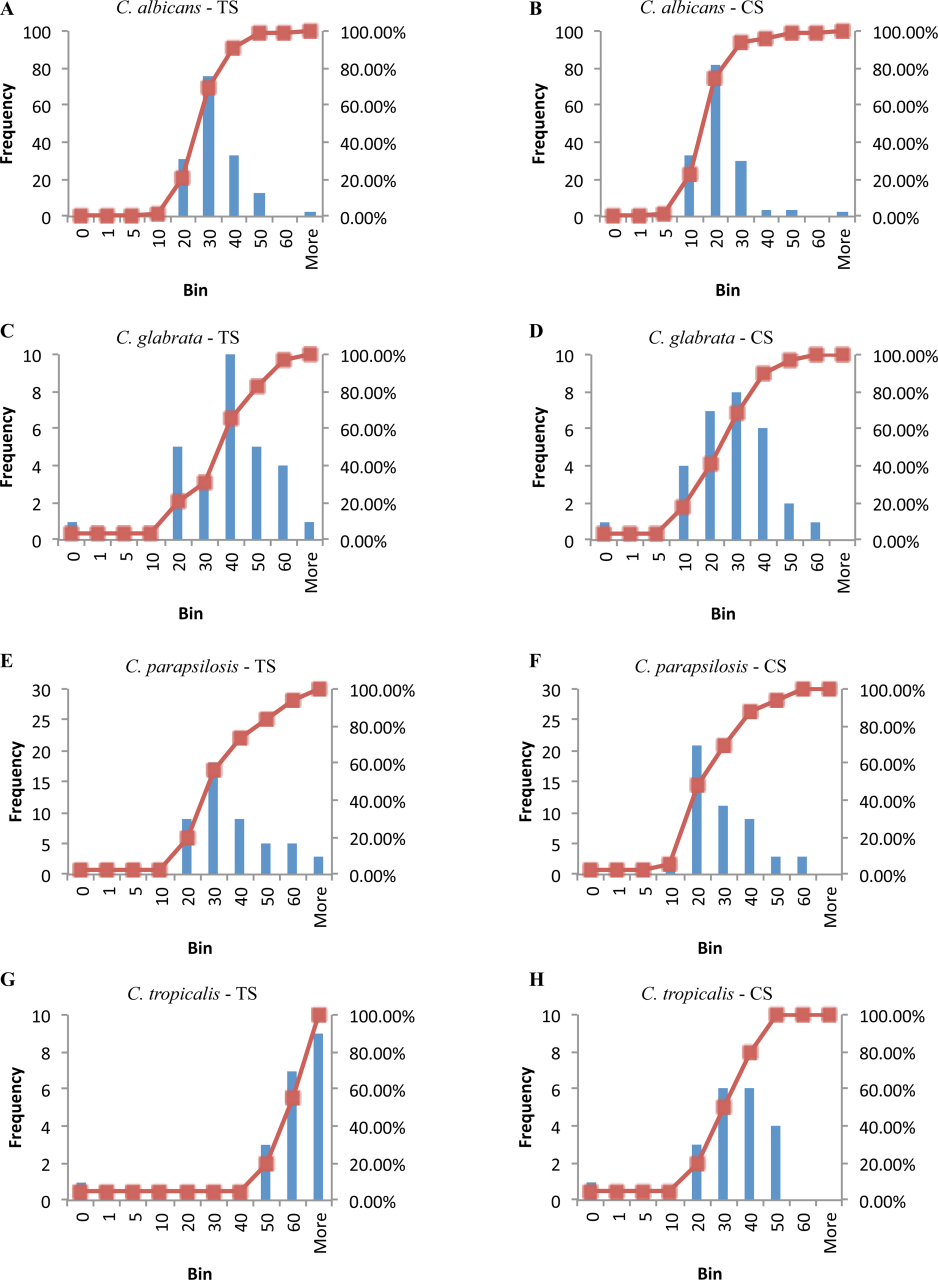
Distribution of the strains distances to TS (type strain) and CS (central strain). Distribution of strains distances reference spectra of the four *Candida* species respect to TS (A, C, E and G) and CS (B, D, F and H), respectively.

In order to determine distance matrixes for FT-IR spectra, distances between all strains were estimated on the basis of PLS scores. To this purpose a PLSDA model was established and the optimal number of components was estimated according to the procedure described above. The following distances were estimated: (1) the distances of all strains to the Type Strain (TS), which is used as a reference strain in NGS approach and (2) the distances of all strains to the Central Strain (CS), which was previously demonstrated to be the optimal reference strain, when a distance approach is used to species delimitation and identification [61] (Fig 6).

The distances of the strains of each species from the CS and the taxonomic TS showed different distributions for each of the four species. In general, the vast majority of the strains showed a short distances to the CS of the PLS distribution rather than the TS. For *C*. *tropicalis* for example, 40% of the strains were distributed around the CS, while the majority of them showed huge distances to the TS. The fact that TS is not central in this case could be due to the small number of strains within the *C*. *tropicalis* subset (19 strains) as well as an overlapping of the species *C*. *tropicalis* with *C*. *parapsilosis* in the block 5 of the NGS-based distance matrix (Fig 3). Also the *C*. *albicans* subset with more than 150 strains did not reveal a central positioning of the type strain in the strain distribution; similar observations were made for *C*. *parapsilosis* and *C*. *glabrata*.

### Taxonomic usage of PLS modeled FT-IR data

The rationale in assigning an unknown strain to a species by phenotypic approaches is that the shortest the distance and the closest will be the microorganisms to that species. Since every species is represented by several strains, one option is to define a single reference strain and determine the distance of an unknown strain to this reference strain. From a taxonomic point of view, the reference strain should be represented by the type strain (TS). Notwithstanding, if the type strain is far from the centre of the strain distribution in a given species, the use of the type strain as reference strain may result in miss-identifications.

For these reasons, we compared the outputs of the identifications carried out with both the type and the central strain. Two procedures based on a single possible match and on two matches were testes, respectively. The former requires that the distance of a strain to the TS or the CS of its species is the shortest among the distances to the TSs and CSs of all species considered. The latter requires that the distance to the TS of the species is the shortest among all distances to the various TSs. The CSs distances are calculated in a similar way.

**Fig 7:**
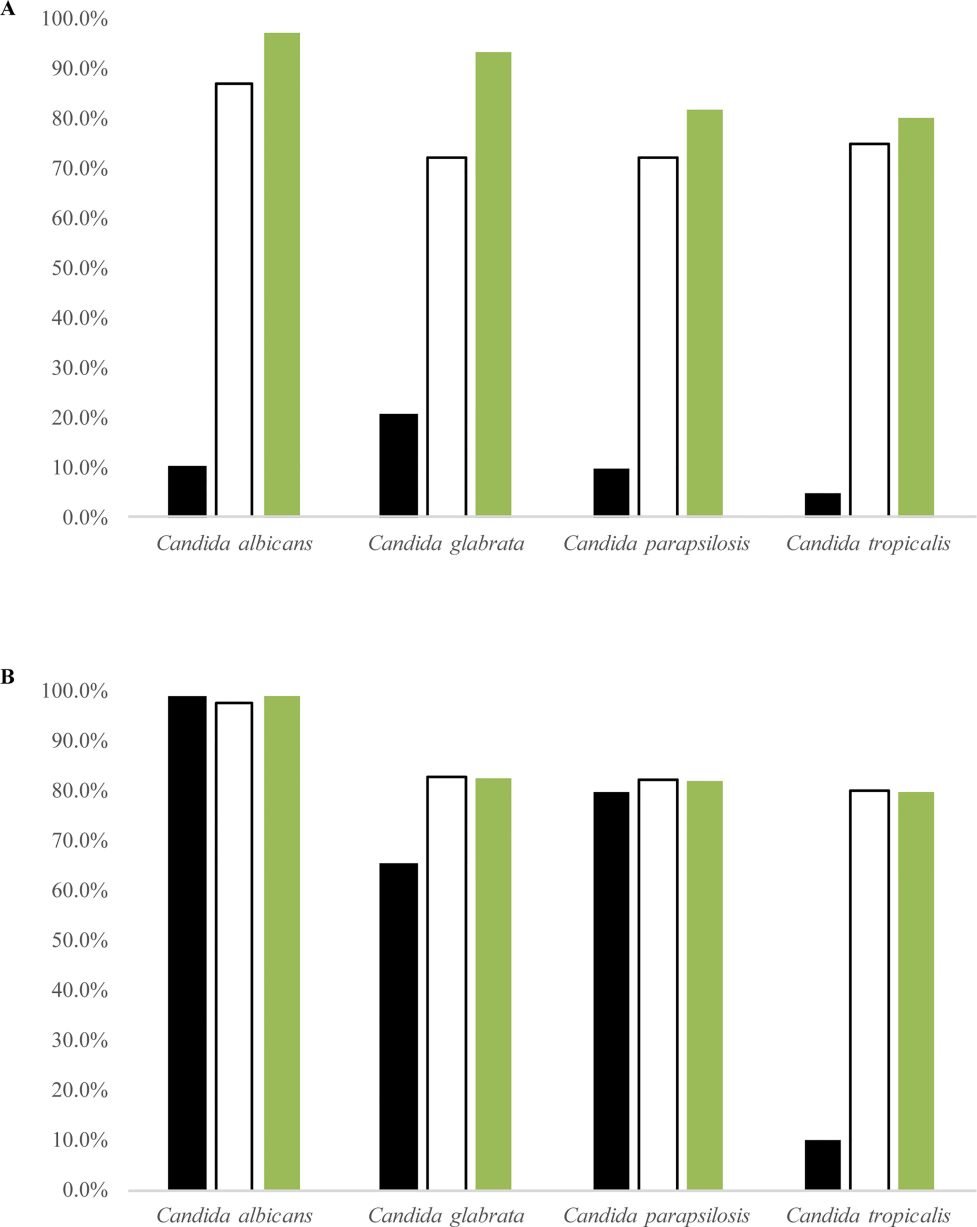
Comparison of single and double match approaches in classify *Candida* strains. (A) Single match analysis with CHROMagar. (B) Double match analysis with CHROMagar and MALDI-TOF. Black columns represent the percentage of matchings’ to the Type Strain (TS); white columns report the matchings’ to the central strain (CS). Green columns report the percentage of the sum of matchings’ in the single match analysis (A) and the maximum obtainable percentage of correct matchings’ in the double match analysis (B).

With the single match procedure, the number of correct identifications was higher using CS than TS (Fig 7A).

Summing the results of both the TS and the CS distances obtained with the single match procedure, the total percentage of correct identification reached 97.4% for *C*. *albicans*, 93.1% for *C*. *glabrata*, while *C*. *parapsilosis* and *C*. *tropicalis* achieved respectively 82% and 80% correct identifications. With the double match procedure, the successful identification to the TS and CS were very similar for *C*. *albicans* and *C*. *parapsilosis* (Fig 7B). The other two species showed lower identification rates with the double matching procedure. Interestingly, in *C*. *albicans* 97.4% of the strains were correctly identified with two matches. The proportion of strains with two matches decreased to 74% in *C*. *parapsilosis*, 62.1% in *C*. *glabrata* and was only 15% in *C*. *tropicalis*.

A possible interpretation of those results was that the identification success could be due to the number of strains actually used in the PLS analysis. Therefore, we investigated the correlation between the number of correct identifications and the number of strains in a given species. In the single match case, the correlation between the number of strains and the percentage of positive identifications was 0.7060. This poor correlation value, sometimes resulting even in lower value when all strains were considered, can be probably ascribed to the fact that *C*. *glabrata* showed more matching that expected. Considering only three species *C*. *albicans*, *C*. *parapsilosis* and *C*. *tropicalis*, the correlation between the number of correctly identified strains and the number of strains in each species resulted 0.9943. For the double match algorithm, the correlation between the number of strains and the percentage of correct identification was 0.9849 when all species were included. These results demonstrate that the quality of the identification depends strongly on the number of correctly identified reference strains used to create the PLS model [34, 44].

## Discussion

Taxonomy of fungi is subject to the code of nomenclature (http://www.iapt-taxon.org/nomen/main.php), requiring that “The application of names of taxonomic groups is determined by means of nomenclatural types” (Principle II). The type is defined in the article 7.2 as follows:”A nomenclatural type (*typus*) is that element to which the name of a taxon is permanently attached, whether as the correct name or as a synonym. The nomenclatural type is not necessarily the most typical or representative element of a taxon”. A living “type strain” represents the type in microbiology. The fact that the type strain is not necessarily the most representative, poses serious problems, when a distance-based approach is applied for the classification of unknown strain. In fact, it was demonstrated that serious problems in identification could be due to reference strains far from the centre of the strain distribution, extreme closeness of the species and width of their distribution. The worst situation is present when two or more species are closer than their mean variation.

Recent papers demonstrated that the type strain is central in many species when using the ITS as taxonomic marker. The same situation was mostly present when the analysis was focused on fungal species of medical interest. For the four species considered in this paper, the type strain was not central. We have shown that the quality of the identification depends very strongly on the number of strains employed with an acceptable minimum of at least 50, as in the case of *C*. *parapsilosis*. Even *C*. *albicans*, with more than 150 strains, did not show the centrality of type strain in the strain distribution. Taking into account these considerations, one may hypothesize that increasing the number of strains leads to an improvement of identification quality, but it is unlikely that the type strain converges towards the centre of the PLS distribution. These evidences raise the question for the rational of this phenomenon and pose a practical problem related to the correct identification procedure using FT-IR technology.

The rationale behind those differences can be due to the strong independence between the FT-IR metabolomics and the ITS-LSU D1/D2 description of the strains. In fact, there is no evidence that the metabolome and the sequence of these DNA markers should be biologically linked. On the other hand, the evolutionary divergence between species can have varied at similar pace for both DNA markers and metabolome, making the two systems comparable, although not biologically dependent as, for instance, a protein sequence with the DNA sequence of its encoding gene.

Reliable identifications at species level are obtained routinely with MALDI-TOF. Still, FT-IR identification of microbial strains could be of general and medical interest for at least two factors: *i*. lower costs of FT-IR vs. MALDI-TOF and *ii*. Possibility to type strains with FT-IR. The former reason is mandatory for small institutions that could afford and FTIR-HTS apparatus but not the MALDI-TOF. Moreover, the availability of reliable and portable FT-IR devices is a further opportunity of application at low instrument cost. Strain typing with FT-IR has been demonstrated in a number of studies [62], whereas the possibility offered by MALDI-TOF for this operation are still matter of an active investigation [63].

From a practical point of view, it seems that the right procedure to employ FT-IR as an effective system in strain identification relies on three major points: i. a large database of reference spectra of strains identified correctly with state of the art methods, ii. an efficient statistical modeling, iii. the simultaneous use of the central and type strain with *ad hoc* tailored algorithms as those described in this paper or more advanced algorithms based on pattern recognition [61].

The data shown indicate that when high numbers of strains are considered, the lack of centrality of the type strain plays a secondary role. Moreover, the application of the double match algorithm allows for a more careful identification. In fact, strains scoring “1” should be double checked with other analyses, whereas the identification of strains scoring “2” matching can be considered highly satisfactory.

As compared to other high-throughput techniques such as NGS, FT-IR has several advantages; included easy and fast sample preparation as well as low costs for consumables, which makes this spectroscopic technique very attractive and suitable for medical diagnostics. However, the current limitation to its use seems to be the absence of reliable and validated libraries linked to taxonomically sound identification procedure. In principle, libraries should include several tens of strains for each relevant species, possibly over 50, according to our data. At the same time, the panel of strains needs to be composed of well-identified strains, possibly deriving from diverse sources and collected over an extensive time period. This implies a multidisciplinary effort of specialists working in strain isolation and maintenance, molecular taxonomy, FT-IR technique and chemo-metrics, data management and data basing.

## Supporting information

S1 Table 256 strains employed in the study. (DOCX)

S2 Table GenBank accession numbers of 256 consensus sequences. (DOCX)

S3 Table Percentages of identity and coverage of 256 consensus sequences derived from global pairwise alignment. (DOCX)
