# Supplementary material for: Merging FT-IR and NGS for simultaneous phenotypic and genotypic identification of pathogenic Candida species
Source: PLoS One. 2017 Dec 4;12(12):e0188104. doi: 10.1371/journal.pone.0188104 (PMC5714347; doi:10.1371/journal.pone.0188104)
Supplement: S1 Table — (DOCX) [file pone.0188104.s001.docx]

| **Strain** | **Species** | **Ward** | **City** | **Strain** | **Species** | **Ward** | **City** |
| --- | --- | --- | --- | --- | --- | --- | --- |
| **Number** |  |  |  | **Number** |  |  |  |
| **CMC 1965** | *C. albicans* | Sp. Medicine | Pi | **CMC 1913** | *C. albicans* | Gen. Medicine | Ud |
| **CMC 1966** | *C. albicans* | Sp. Medicine | Pi | **CMC 1914** | *C. albicans* | Surgery | Ud |
| **CMC 1969** | *C. albicans* | Sp. Medicine | Pi | **CMC 1915** | *C. albicans* | Sp. Medicine | Ud |
| **CMC 1970** | *C. albicans* | Sp. Medicine | Pi | **CMC 1918** | *C. albicans* | Rehabilitation | Ud |
| **CMC 1974** | *C. albicans* | Sp. Medicine | Pi | **CMC 1919** | *C. albicans* | Gen. Medicine | Ud |
| **CMC 1977** | *C. albicans* | Sp. Medicine | Pi | **CMC 1920** | *C. albicans* | Gen. Medicine | Ud |
| **CMC 1980** | *C. albicans* | Surgery | Pi | **CMC 1921** | *C. albicans* | Gen. Medicine | Ud |
| **CMC 1982** | *C. albicans* | Sp. Medicine | Pi | **CMC 1923** | *C. albicans* | Gen. Medicine | Ud |
| **CMC 1983** | *C. albicans* | ICU | Pi | **CMC 1925** | *C. albicans* | Sp. Medicine | Ud |
| **CMC 1985** | *C. albicans* | Sp. Medicine | Pi | **CMC 1926** | *C. albicans* | Gen. Medicine | Ud |
| **CMC 1986** | *C. albicans* | ICU | Pi | **CMC 1927** | *C. albicans* | Surgery | Ud |
| **CMC 1987** | *C. albicans* | Surgery | Pi | **CMC 1928** | *C. albicans* | Sp. Medicine | Ud |
| **CMC 1988** | *C. albicans* | Surgery | Pi | **CMC 1931** | *C. albicans* | Surgery | Ud |
| **CMC 1990** | *C. albicans* | ICU | Pi | **CMC 1932** | *C. albicans* | Gen. Medicine | Ud |
| **CMC 1991** | *C. albicans* | Surgery | Pi | **CMC 1936** | *C. albicans* | Sp. Medicine | Ud |
| **CMC 1992** | *C. albicans* | ICU | Pi | **CMC 1937** | *C. albicans* | Gen. Medicine | Ud |
| **CMC 1994** | *C. albicans* | Sp. Medicine | Pi | **CMC 1940** | *C. albicans* | Gen. Medicine | Ud |
| **CMC 1998** | *C. albicans* | ICU | Pi | **CMC 1941** | *C. albicans* | Gen. Medicine | Ud |
| **CMC 2000** | *C. albicans* | Sp. Medicine | Pi | **CMC 1942** | *C. albicans* | Surgery | Ud |
| **CMC 2001** | *C. albicans* | Sp. Medicine | Pi | **CMC 1946** | *C. albicans* | Sp. Medicine | Ud |
| **CMC 2008** | *C. albicans* | Sp. Medicine | Pi | **CMC 1952** | *C. albicans* | Gen. Medicine | Ud |
| **CMC 2019** | *C. albicans* | ICU | Pi | **CMC 1954** | *C. albicans* | Surgery | Ud |
| **CMC 2020** | *C. albicans* | Surgery | Pi | **CMC 1957** | *C. albicans* | Gen. Medicine | Ud |
| **CMC 2023** | *C. albicans* | Gen. Medicine | Pi | **CMC 1958** | *C. albicans* | Surgery | Ud |
| **CMC 2025** | *C. albicans* | ICU | Pi | **CMC 1959** | *C. albicans* | Surgery | Ud |
| **CMC 2026** | *C. albicans* | Surgery | Pi | **CMC 1960** | *C. albicans* | Gen. Medicine | Ud |
| **CMC 2029** | *C. albicans* | Sp. Medicine | Pi | **CMC 1962** | *C. albicans* | Gen. Medicine | Ud |
| **CMC 2030** | *C. albicans* | ICU | Pi | **CMC 1963** | *C. albicans* | Rehabilitation | Ud |
| **CMC 2031** | *C. albicans* | Surgery | Pi | **CMC 1976** | *C. glabrata* | Sp. Medicine | Pi |
| **CMC 2033** | *C. albicans* | Surgery | Pi | **CMC 1989** | *C. glabrata* | ICU | Pi |
| **CMC 2034** | *C. albicans* | Gen. Medicine | Pi | **CMC 2007** | *C. glabrata* | Sp. Medicine | Pi |
| **CMC 2035** | *C. albicans* | Sp. Medicine | Pi | **CMC 2015** | *C. glabrata* | Gen. Medicine | Pi |
| **CMC 2036** | *C. albicans* | Surgery | Pi | **CMC 2018** | *C. glabrata* | ICU | Pi |
| **CMC 2037** | *C. albicans* | Gen. Medicine | Pi | **CMC 2027** | *C. glabrata* | Surgery | Pi |
| **CMC 2042** | *C. albicans* | ICU | Pi | **CMC 2032** | *C. glabrata* | Surgery | Pi |
| **CMC 2043** | *C. albicans* | Gen. Medicine | Pi | **CMC 1782** | *C. glabrata* | ICU | Ud |
| **CMC 2045** | *C. albicans* | Sp. Medicine | Pi | **CMC 1807** | *C. glabrata* | Gen. Medicine | Ud |
| **CMC 2046** | *C. albicans* | ICU | Pi | **CMC 1813** | *C. glabrata* | Gen. Medicine | Ud |
| **CMC 2048** | *C. albicans* | Sp. Medicine | Pi | **CMC 1817** | *C. glabrata* | Gen. Medicine | Ud |
| **CMC 2049** | *C. albicans* | Surgery | Pi | **CMC 1830** | *C. glabrata* | Surgery | Ud |
| **CMC 2053** | *C. albicans* | Sp. Medicine | Pi | **CMC 1832** | *C. glabrata* | Oncohematology | Ud |
| **CMC 1768** | *C. albicans* | Surgery | Ud | **CMC 1837** | *C. glabrata* | Gen. Medicine | Ud |
| **CMC 1769** | *C. albicans* | Gen. Medicine | Ud | **CMC 1846** | *C. glabrata* | Gen. Medicine | Ud |
| **CMC 1770** | *C. albicans* | Gen. Medicine | Ud | **CMC 1857** | *C. glabrata* | Gen. Medicine | Ud |
| **CMC 1771** | *C. albicans* | Gen. Medicine | Ud | **CMC 1860** | *C. glabrata* | Gen. Medicine | Ud |
| **CMC 1773** | *C. albicans* | Gen. Medicine | Ud | **CMC 1861** | *C. glabrata* | Gen. Medicine | Ud |
| **CMC 1774** | *C. albicans* | ICU | Ud | **CMC 1864** | *C. glabrata* | ICU | Ud |
| **CMC 1776** | *C. albicans* | Gen. Medicine | Ud | **CMC 1865** | *C. glabrata* | Surgery | Ud |
| **CMC 1777** | *C. albicans* | ICU | Ud | **CMC 1884** | *C. glabrata* | Gen. Medicine | Ud |
| **CMC 1778** | *C. albicans* | Sp. Medicine | Ud | **CMC 1894** | *C. glabrata* | Rehabilitation | Ud |
| **CMC 1780** | *C. albicans* | Gen. Medicine | Ud | **CMC 1895** | *C. glabrata* | Surgery | Ud |
| **CMC 1785** | *C. albicans* | Sp. Medicine | Ud | **CMC 1912** | *C. glabrata* | Gen. Medicine | Ud |
| **CMC 1786** | *C. albicans* | Sp. Medicine | Ud | **CMC 1916** | *C. glabrata* | ICU | Ud |
| **CMC 1788** | *C. albicans* | Surgery | Ud | **CMC 1933** | *C. glabrata* | Gen. Medicine | Ud |
| **CMC 1790** | *C. albicans* | Gen. Medicine | Ud | **CMC 1934** | *C. glabrata* | Surgery | Ud |
| **CMC 1794** | *C. albicans* | Surgery | Ud | **CMC 1938** | *C. glabrata* | Gen. Medicine | Ud |
| **CMC 1795** | *C. albicans* | Gen. Medicine | Ud | **CMC 1950** | *C. glabrata* | Sp. Medicine | Ud |
| **CMC 1797** | *C. albicans* | Oncohematology | Ud | **CMC 1964** | *C. glabrata* | Sp. Medicine | Ud |
| **CMC 1799** | *C. albicans* | ICU | Ud | **CMC 1972** | *C. parapsilosis* | Sp. Medicine | Pi |
| **CMC 1802** | *C. albicans* | ICU | Ud | **CMC 1979** | *C. parapsilosis* | ICU | Pi |
| **CMC 1803** | *C. albicans* | ICU | Ud | **CMC 1973** | *C. parapsilosis* | Surgery | Pi |
| **CMC 1804** | *C. albicans* | Surgery | Ud | **CMC 1981** | *C. parapsilosis* | Sp. Medicine | Pi |
| **CMC 1806** | *C. albicans* | Surgery | Ud | **CMC 2006** | *C. parapsilosis* | ICU | Pi |
| **CMC 1811** | *C. albicans* | Gen. Medicine | Ud | **CMC 2012** | *C. parapsilosis* | ICU | Pi |
| **CMC 1815** | *C. albicans* | Gen. Medicine | Ud | **CMC 2013** | *C. parapsilosis* | Sp. Medicine | Pi |
| **CMC 1816** | *C. albicans* | Sp. Medicine | Ud | **CMC 2014** | *C. parapsilosis* | Sp. Medicine | Pi |
| **CMC 1818** | *C. albicans* | Gen. Medicine | Ud | **CMC 2016** | *C. parapsilosis* | Sp. Medicine | Pi |
| **CMC 1819** | *C. albicans* | Surgery | Ud | **CMC 2022** | *C. parapsilosis* | Surgery | Pi |
| **CMC 1820** | *C. albicans* | Sp. Medicine | Ud | **CMC 2038** | *C. parapsilosis* | Sp. Medicine | Pi |
| **CMC 1821** | *C. albicans* | Surgery | Ud | **CMC 2039** | *C. parapsilosis* | Surgery | Pi |
| **CMC 1822** | *C. albicans* | Surgery | Ud | **CMC 2040** | *C. parapsilosis* | Surgery | Pi |
| **CMC 1823** | *C. albicans* | Gen. Medicine | Ud | **CMC 2044** | *C. parapsilosis* | ICU | Pi |
| **CMC 1824** | *C. albicans* | Surgery | Ud | **CMC 2050** | *C. parapsilosis* | Sp. Medicine | Pi |
| **CMC 1828** | *C. albicans* | Surgery | Ud | **CMC 1772** | *C. parapsilosis* | Sp. Medicine | Ud |
| **CMC 1829** | *C. albicans* | Sp. Medicine | Ud | **CMC 1781** | *C. parapsilosis* | Oncohematology | Ud |
| **CMC 1831** | *C. albicans* | Surgery | Ud | **CMC 1783** | *C. parapsilosis* | Gen. Medicine | Ud |
| **CMC 1833** | *C. albicans* | Surgery | Ud | **CMC 1787** | *C. parapsilosis* | Gen. Medicine | Ud |
| **CMC 1834** | *C. albicans* | Gen. Medicine | Ud | **CMC 1791** | *C. parapsilosis* | Gen. Medicine | Ud |
| **CMC 1835** | *C. albicans* | Sp. Medicine | Ud | **CMC 1792** | *C. parapsilosis* | ICU | Ud |
| **CMC 1840** | *C. albicans* | Surgery | Ud | **CMC 1793** | *C. parapsilosis* | Gen. Medicine | Ud |
| **CMC 1842** | *C. albicans* | Surgery | Ud | **CMC 1796** | *C. parapsilosis* | Sp. Medicine | Ud |
| **CMC 1843** | *C. albicans* | Oncohematology | Ud | **CMC 1800** | *C. parapsilosis* | Sp. Medicine | Ud |
| **CMC 1844** | *C. albicans* | Gen. Medicine | Ud | **CMC 1801** | *C. parapsilosis* | Sp. Medicine | Ud |
| **CMC 1845** | *C. albicans* | Gen. Medicine | Ud | **CMC 1805** | *C. parapsilosis* | Gen. Medicine | Ud |
| **CMC 1847** | *C. albicans* | Gen. Medicine | Ud | **CMC 1808** | *C. parapsilosis* | Gen. Medicine | Ud |
| **CMC 1848** | *C. albicans* | Sp. Medicine | Ud | **CMC 1809** | *C. parapsilosis* | Gen. Medicine | Ud |
| **CMC 1849** | *C. albicans* | Sp. Medicine | Ud | **CMC 1812** | *C. parapsilosis* | Gen. Medicine | Ud |
| **CMC 1850** | *C. albicans* | ICU | Ud | **CMC 1814** | *C. parapsilosis* | Oncohematology | Ud |
| **CMC 1852** | *C. albicans* | Gen. Medicine | Ud | **CMC 1826** | *C. parapsilosis* | Gen. Medicine | Ud |
| **CMC 1853** | *C. albicans* | Gen. Medicine | Ud | **CMC 1838** | *C. parapsilosis* | Gen. Medicine | Ud |
| **CMC 1854** | *C. albicans* | Sp. Medicine | Ud | **CMC 1841** | *C. parapsilosis* | Surgery | Ud |
| **CMC 1856** | *C. albicans* | ICU | Ud | **CMC 1851** | *C. parapsilosis* | Sp. Medicine | Ud |
| **CMC 1858** | *C. albicans* | Gen. Medicine | Ud | **CMC 1859** | *C. parapsilosis* | Sp. Medicine | Ud |
| **CMC 1862** | *C. albicans* | Gen. Medicine | Ud | **CMC 1867** | *C. parapsilosis* | ICU | Ud |
| **CMC 1863** | *C. albicans* | Gen. Medicine | Ud | **CMC 1880** | *C. parapsilosis* | Sp. Medicine | Ud |
| **CMC 1866** | *C. albicans* | Surgery | Ud | **CMC 1892** | *C. parapsilosis* | Rehabilitation | Ud |
| **CMC 1868** | *C. albicans* | Gen. Medicine | Ud | **CMC 1899** | *C. parapsilosis* | Gen. Medicine | Ud |
| **CMC 1869** | *C. albicans* | Sp. Medicine | Ud | **CMC 1909** | *C. parapsilosis* | Sp. Medicine | Ud |
| **CMC 1870** | *C. albicans* | Sp. Medicine | Ud | **CMC 1922** | *C. parapsilosis* | Gen. Medicine | Ud |
| **CMC 1871** | *C. albicans* | Sp. Medicine | Ud | **CMC 1902** | *C. parapsilosis* | Gen. Medicine | Ud |
| **CMC 1872** | *C. albicans* | Sp. Medicine | Ud | **CMC 1917** | *C. parapsilosis* | Gen. Medicine | Ud |
| **CMC 1873** | *C. albicans* | Gen. Medicine | Ud | **CMC 1929** | *C. parapsilosis* | ICU | Ud |
| **CMC 1875** | *C. albicans* | Gen. Medicine | Ud | **CMC 1930** | *C. parapsilosis* | Sp. Medicine | Ud |
| **CMC 1876** | *C. albicans* | Gen. Medicine | Ud | **CMC 1935** | *C. parapsilosis* | Gen. Medicine | Ud |
| **CMC 1877** | *C. albicans* | ICU | Ud | **CMC 1939** | *C. parapsilosis* | Surgery | Ud |
| **CMC 1878** | *C. albicans* | Gen. Medicine | Ud | **CMC 1945** | *C. parapsilosis* | Oncohematology | Ud |
| **CMC 1879** | *C. albicans* | Gen. Medicine | Ud | **CMC 1948** | *C. parapsilosis* | Sp. Medicine | Ud |
| **CMC 1881** | *C. albicans* | Gen. Medicine | Ud | **CMC 1949** | *C. parapsilosis* | Gen. Medicine | Ud |
| **CMC 1885** | *C. albicans* | Surgery | Ud | **CMC 1951** | *C. parapsilosis* | Sp. Medicine | Ud |
| **CMC 1886** | *C. albicans* | Gen. Medicine | Ud | **CMC 1978** | *C. tropicalis* | Sp. Medicine | Pi |
| **CMC 1887** | *C. albicans* | ICU | Ud | **CMC 2003** | *C. tropicalis* | Sp. Medicine | Pi |
| **CMC 1888** | *C. albicans* | Sp. Medicine | Ud | **CMC 2009** | *C. tropicalis* | Sp. Medicine | Pi |
| **CMC 1889** | *C. albicans* | Gen. Medicine | Ud | **CMC 2017** | *C. tropicalis* | Sp. Medicine | Pi |
| **CMC 1890** | *C. albicans* | Gen. Medicine | Ud | **CMC 2024** | *C. tropicalis* | ICU | Pi |
| **CMC 1891** | *C. albicans* | Sp. Medicine | Ud | **CMC 2041** | *C. tropicalis* | Gen. Medicine | Pi |
| **CMC 1893** | *C. albicans* | Oncohematology | Ud | **CMC 2052** | *C. tropicalis* | Sp. Medicine | Pi |
| **CMC 1896** | *C. albicans* | Gen. Medicine | Ud | **CMC 1784** | *C. tropicalis* | Oncohematology | Ud |
| **CMC 1897** | *C. albicans* | Rehabilitation | Ud | **CMC 1798** | *C. tropicalis* | Gen. Medicine | Ud |
| **CMC 1898** | *C. albicans* | Gen. Medicine | Ud | **CMC 1810** | *C. tropicalis* | Oncohematology | Ud |
| **CMC 1900** | *C. albicans* | Gen. Medicine | Ud | **CMC 1827** | *C. tropicalis* | Gen. Medicine | Ud |
| **CMC 1901** | *C. albicans* | Gen. Medicine | Ud | **CMC 1836** | *C. tropicalis* | Gen. Medicine | Ud |
| **CMC 1903** | *C. albicans* | Gen. Medicine | Ud | **CMC 1839** | *C. tropicalis* | Gen. Medicine | Ud |
| **CMC 1905** | *C. albicans* | Gen. Medicine | Ud | **CMC 1855** | *C. tropicalis* | Oncohematology | Ud |
| **CMC 1906** | *C. albicans* | Gen. Medicine | Ud | **CMC 1874** | *C. tropicalis* | Gen. Medicine | Ud |
| **CMC 1907** | *C. albicans* | Gen. Medicine | Ud | **CMC 1904** | *C. tropicalis* | Sp. Medicine | Ud |
| **CMC 1908** | *C. albicans* | ICU | Ud | **CMC 1953** | *C. tropicalis* | Oncohematology | Ud |
| **CMC 1910** | *C. albicans* | ICU | Ud | **CMC 1956** | *C. tropicalis* | Gen. Medicine | Ud |
| **CMC 1911** | *C. albicans* | Gen. Medicine | Ud | **CMC 1961** | *C. tropicalis* | Gen. Medicine | Ud |
