# Supplementary material for: Merging FT-IR and NGS for simultaneous phenotypic and genotypic identification of pathogenic Candida species
Source: PLoS One. 2017 Dec 4;12(12):e0188104. doi: 10.1371/journal.pone.0188104 (PMC5714347; doi:10.1371/journal.pone.0188104)
Supplement: S2 Table — (DOCX) [file pone.0188104.s002.docx]

| **Strain** | **Species** | **GB Number** | **Strain** | **Species** | **GB Number** | **Strain** | **Species** | **GB Number** |
| --- | --- | --- | --- | --- | --- | --- | --- | --- |
| **Number** |  |  | **Number** |  |  | **Number** |  |  |
| **CMC 1809** | *C. parapsilosis* | MF767623 | **CMC 1932** | *C. albicans* | MF767709 | **CMC 1835** | *C. albicans* | MF767795 |
| **CMC 1805** | *C. parapsilosis* | MF767624 | **CMC 1896** | *C. albicans* | MF767710 | **CMC 1774** | *C. albicans* | MF767796 |
| **CMC 1791** | *C. parapsilosis* | MF767625 | **CMC 1966** | *C. albicans* | MF767711 | **CMC 1887** | *C. albicans* | MF767797 |
| **CMC 2013** | *C. parapsilosis* | MF767626 | **CMC 1893** | *C. albicans* | MF767712 | **CMC 1869** | *C. albicans* | MF767798 |
| **CMC 2044** | *C. parapsilosis* | MF767627 | **CMC 2037** | *C. albicans* | MF767713 | **CMC 1771** | *C. albicans* | MF767799 |
| **CMC 1979** | *C. parapsilosis* | MF767628 | **CMC 1879** | *C. albicans* | MF767714 | **CMC 1840** | *C. albicans* | MF767800 |
| **CMC 1814** | *C. parapsilosis* | MF767629 | **CMC 1881** | *C. albicans* | MF767715 | **CMC 1873** | *C. albicans* | MF767801 |
| **CMC 1793** | *C. parapsilosis* | MF767630 | **CMC 2019** | *C. albicans* | MF767716 | **CMC 1866** | *C. albicans* | MF767802 |
| **CMC 1801** | *C. parapsilosis* | MF767631 | **CMC 1900** | *C. albicans* | MF767717 | **CMC 1886** | *C. albicans* | MF767803 |
| **CMC 2014** | *C. parapsilosis* | MF767632 | **CMC 1868** | *C. albicans* | MF767718 | **CMC 1970** | *C. albicans* | MF767804 |
| **CMC 1783** | *C. parapsilosis* | MF767633 | **CMC 1820** | *C. albicans* | MF767719 | **CMC 1852** | *C. albicans* | MF767805 |
| **CMC 2006** | *C. parapsilosis* | MF767634 | **CMC 1965** | *C. albicans* | MF767720 | **CMC 1834** | *C. albicans* | MF767806 |
| **CMC 2040** | *C. parapsilosis* | MF767635 | **CMC 1990** | *C. albicans* | MF767721 | **CMC 1785** | *C. albicans* | MF767807 |
| **CMC 1981** | *C. parapsilosis* | MF767636 | **CMC 1921** | *C. albicans* | MF767722 | **CMC 2000** | *C. albicans* | MF767808 |
| **CMC 2016** | *C. parapsilosis* | MF767637 | **CMC 1974** | *C. albicans* | MF767723 | **CMC 1911** | *C. albicans* | MF767809 |
| **CMC 1851** | *C. parapsilosis* | MF767638 | **CMC 1977** | *C. albicans* | MF767724 | **CMC 1969** | *C. albicans* | MF767810 |
| **CMC 1796** | *C. parapsilosis* | MF767639 | **CMC 1875** | *C. albicans* | MF767725 | **CMC 1769** | *C. albicans* | MF767811 |
| **CMC 2022** | *C. parapsilosis* | MF767640 | **CMC 2049** | *C. albicans* | MF767726 | **CMC 1962** | *C. albicans* | MF767812 |
| **CMC 1909** | *C. parapsilosis* | MF767641 | **CMC 1831** | *C. albicans* | MF767727 | **CMC 1788** | *C. albicans* | MF767813 |
| **CMC 1841** | *C. parapsilosis* | MF767642 | **CMC 1885** | *C. albicans* | MF767728 | **CMC 1920** | *C. albicans* | MF767814 |
| **CMC 1838** | *C. parapsilosis* | MF767643 | **CMC 2042** | *C. albicans* | MF767729 | **CMC 1824** | *C. albicans* | MF767815 |
| **CMC 1772** | *C. parapsilosis* | MF767644 | **CMC 1958** | *C. albicans* | MF767730 | **CMC 1914** | *C. albicans* | MF767816 |
| **CMC 1922** | *C. parapsilosis* | MF767645 | **CMC 1858** | *C. albicans* | MF767731 | **CMC 1897** | *C. albicans* | MF767817 |
| **CMC 1812** | *C. parapsilosis* | MF767646 | **CMC 1946** | *C. albicans* | MF767732 | **CMC 1906** | *C. albicans* | MF767818 |
| **CMC 1867** | *C. parapsilosis* | MF767647 | **CMC 2046** | *C. albicans* | MF767733 | **CMC 1913** | *C. albicans* | MF767819 |
| **CMC 1859** | *C. parapsilosis* | MF767648 | **CMC 1853** | *C. albicans* | MF767734 | **CMC 1833** | *C. albicans* | MF767820 |
| **CMC 2039** | *C. parapsilosis* | MF767649 | **CMC 1845** | *C. albicans* | MF767735 | **CMC 1926** | *C. albicans* | MF767821 |
| **CMC 1973** | *C. parapsilosis* | MF767650 | **CMC 1829** | *C. albicans* | MF767736 | **CMC 1898** | *C. albicans* | MF767822 |
| **CMC 1880** | *C. parapsilosis* | MF767651 | **CMC 1963** | *C. albicans* | MF767737 | **CMC 1928** | *C. albicans* | MF767823 |
| **CMC 1945** | *C. parapsilosis* | MF767652 | **CMC 1987** | *C. albicans* | MF767738 | **CMC 1862** | *C. albicans* | MF767824 |
| **CMC 1902** | *C. parapsilosis* | MF767653 | **CMC 1889** | *C. albicans* | MF767739 | **CMC 1849** | *C. albicans* | MF767825 |
| **CMC 1930** | *C. parapsilosis* | MF767654 | **CMC 1910** | *C. albicans* | MF767740 | **CMC 1890** | *C. albicans* | MF767826 |
| **CMC 1787** | *C. parapsilosis* | MF767655 | **CMC 1770** | *C. albicans* | MF767741 | **CMC 1803** | *C. albicans* | MF767827 |
| **CMC 2012** | *C. parapsilosis* | MF767656 | **CMC 1931** | *C. albicans* | MF767742 | **CMC 1888** | *C. albicans* | MF767828 |
| **CMC 1826** | *C. parapsilosis* | MF767657 | **CMC 2043** | *C. albicans* | MF767743 | **CMC 1815** | *C. albicans* | MF767829 |
| **CMC 1935** | *C. parapsilosis* | MF767658 | **CMC 1850** | *C. albicans* | MF767744 | **CMC 2027** | *C. glabrata* | MF767830 |
| **CMC 1808** | *C. parapsilosis* | MF767659 | **CMC 1848** | *C. albicans* | MF767745 | **CMC 2018** | *C. glabrata* | MF767831 |
| **CMC 1800** | *C. parapsilosis* | MF767660 | **CMC 1843** | *C. albicans* | MF767746 | **CMC 1976** | *C. glabrata* | MF767832 |
| **CMC 1929** | *C. parapsilosis* | MF767661 | **CMC 1957** | *C. albicans* | MF767747 | **CMC 2032** | *C. glabrata* | MF767833 |
| **CMC 2038** | *C. parapsilosis* | MF767662 | **CMC 1776** | *C. albicans* | MF767748 | **CMC 2007** | *C. glabrata* | MF767834 |
| **CMC 1948** | *C. parapsilosis* | MF767663 | **CMC 1918** | *C. albicans* | MF767749 | **CMC 1807** | *C. glabrata* | MF767835 |
| **CMC 1951** | *C. parapsilosis* | MF767664 | **CMC 1870** | *C. albicans* | MF767750 | **CMC 1934** | *C. glabrata* | MF767836 |
| **CMC 1939** | *C. parapsilosis* | MF767665 | **CMC 1773** | *C. albicans* | MF767751 | **CMC 1916** | *C. glabrata* | MF767837 |
| **CMC 1892** | *C. parapsilosis* | MF767666 | **CMC 1822** | *C. albicans* | MF767752 | **CMC 1830** | *C. glabrata* | MF767838 |
| **CMC 1972** | *C. parapsilosis* | MF767667 | **CMC 1959** | *C. albicans* | MF767753 | **CMC 1865** | *C. glabrata* | MF767839 |
| **CMC 2050** | *C. parapsilosis* | MF767668 | **CMC 1802** | *C. albicans* | MF767754 | **CMC 1950** | *C. glabrata* | MF767840 |
| **CMC 1917** | *C. parapsilosis* | MF767669 | **CMC 1842** | *C. albicans* | MF767755 | **CMC 1884** | *C. glabrata* | MF767841 |
| **CMC 1949** | *C. parapsilosis* | MF767670 | **CMC 1877** | *C. albicans* | MF767756 | **CMC 1912** | *C. glabrata* | MF767842 |
| **CMC 1781** | *C. parapsilosis* | MF767671 | **CMC 1901** | *C. albicans* | MF767757 | **CMC 1938** | *C. glabrata* | MF767843 |
| **CMC 1899** | *C. parapsilosis* | MF767672 | **CMC 1937** | *C. albicans* | MF767758 | **CMC 1895** | *C. glabrata* | MF767844 |
| **CMC 1792** | *C. parapsilosis* | MF767673 | **CMC 1941** | *C. albicans* | MF767759 | **CMC 1857** | *C. glabrata* | MF767845 |
| **CMC 1799** | *C. albicans* | MF767674 | **CMC 2036** | *C. albicans* | MF767760 | **CMC 1837** | *C. glabrata* | MF767846 |
| **CMC 1811** | *C. albicans* | MF767675 | **CMC 1777** | *C. albicans* | MF767761 | **CMC 1933** | *C. glabrata* | MF767847 |
| **CMC 1794** | *C. albicans* | MF767676 | **CMC 2029** | *C. albicans* | MF767762 | **CMC 1813** | *C. glabrata* | MF767848 |
| **CMC 1797** | *C. albicans* | MF767677 | **CMC 1828** | *C. albicans* | MF767763 | **CMC 1861** | *C. glabrata* | MF767849 |
| **CMC 1804** | *C. albicans* | MF767678 | **CMC 1891** | *C. albicans* | MF767764 | **CMC 1846** | *C. glabrata* | MF767850 |
| **CMC 1844** | *C. albicans* | MF767679 | **CMC 2053** | *C. albicans* | MF767765 | **CMC 1817** | *C. glabrata* | MF767851 |
| **CMC 1823** | *C. albicans* | MF767680 | **CMC 2026** | *C. albicans* | MF767766 | **CMC 1782** | *C. glabrata* | MF767852 |
| **CMC 1907** | *C. albicans* | MF767681 | **CMC 1927** | *C. albicans* | MF767767 | **CMC 1864** | *C. glabrata* | MF767853 |
| **CMC 1806** | *C. albicans* | MF767682 | **CMC 1960** | *C. albicans* | MF767768 | **CMC 1832** | *C. glabrata* | MF767854 |
| **CMC 2048** | *C. albicans* | MF767683 | **CMC 1780** | *C. albicans* | MF767769 | **CMC 1894** | *C. glabrata* | MF767855 |
| **CMC 2001** | *C. albicans* | MF767684 | **CMC 1908** | *C. albicans* | MF767770 | **CMC 1989** | *C. glabrata* | MF767856 |
| **CMC 1942** | *C. albicans* | MF767685 | **CMC 1854** | *C. albicans* | MF767771 | **CMC 1860** | *C. glabrata* | MF767857 |
| **CMC 1903** | *C. albicans* | MF767686 | **CMC 1790** | *C. albicans* | MF767772 | **CMC 1964** | *C. glabrata* | MF767858 |
| **CMC 1985** | *C. albicans* | MF767687 | **CMC 1778** | *C. albicans* | MF767773 | **CMC 2015** | *C. glabrata* | MF767859 |
| **CMC 1795** | *C. albicans* | MF767688 | **CMC 1991** | *C. albicans* | MF767774 | **CMC 2041** | *C. tropicalis* | MF767860 |
| **CMC 1876** | *C. albicans* | MF767689 | **CMC 2030** | *C. albicans* | MF767775 | **CMC 2024** | *C. tropicalis* | MF767861 |
| **CMC 1982** | *C. albicans* | MF767690 | **CMC 2035** | *C. albicans* | MF767776 | **CMC 1978** | *C. tropicalis* | MF767862 |
| **CMC 1819** | *C. albicans* | MF767691 | **CMC 1986** | *C. albicans* | MF767777 | **CMC 2009** | *C. tropicalis* | MF767863 |
| **CMC 1936** | *C. albicans* | MF767692 | **CMC 1992** | *C. albicans* | MF767778 | **CMC 2003** | *C. tropicalis* | MF767864 |
| **CMC 2008** | *C. albicans* | MF767693 | **CMC 1786** | *C. albicans* | MF767779 | **CMC 1961** | *C. tropicalis* | MF767865 |
| **CMC 1980** | *C. albicans* | MF767694 | **CMC 1954** | *C. albicans* | MF767780 | **CMC 1784** | *C. tropicalis* | MF767866 |
| **CMC 1816** | *C. albicans* | MF767695 | **CMC 1994** | *C. albicans* | MF767781 | **CMC 1855** | *C. tropicalis* | MF767867 |
| **CMC 1919** | *C. albicans* | MF767696 | **CMC 1905** | *C. albicans* | MF767782 | **CMC 1836** | *C. tropicalis* | MF767868 |
| **CMC 1818** | *C. albicans* | MF767697 | **CMC 1940** | *C. albicans* | MF767783 | **CMC 2017** | *C. tropicalis* | MF767869 |
| **CMC 1998** | *C. albicans* | MF767698 | **CMC 2045** | *C. albicans* | MF767784 | **CMC 1827** | *C. tropicalis* | MF767870 |
| **CMC 1915** | *C. albicans* | MF767699 | **CMC 1988** | *C. albicans* | MF767785 | **CMC 1798** | *C. tropicalis* | MF767871 |
| **CMC 1856** | *C. albicans* | MF767700 | **CMC 1878** | *C. albicans* | MF767786 | **CMC 1956** | *C. tropicalis* | MF767872 |
| **CMC 2025** | *C. albicans* | MF767701 | **CMC 1847** | *C. albicans* | MF767787 | **CMC 1839** | *C. tropicalis* | MF767873 |
| **CMC 2031** | *C. albicans* | MF767702 | **CMC 1768** | *C. albicans* | MF767788 | **CMC 1874** | *C. tropicalis* | MF767874 |
| **CMC 1983** | *C. albicans* | MF767703 | **CMC 1872** | *C. albicans* | MF767789 | **CMC 2052** | *C. tropicalis* | MF767875 |
| **CMC 1821** | *C. albicans* | MF767704 | **CMC 1925** | *C. albicans* | MF767790 | **CMC 1810** | *C. tropicalis* | MF767876 |
| **CMC 1923** | *C. albicans* | MF767705 | **CMC 1952** | *C. albicans* | MF767791 | **CMC 1953** | *C. tropicalis* | MF767877 |
| **CMC 1871** | *C. albicans* | MF767706 | **CMC 2020** | *C. albicans* | MF767792 | **CMC 1904** | *C. tropicalis* | MF767878 |
| **CMC 2033** | *C. albicans* | MF767707 | **CMC 2023** | *C. albicans* | MF767793 |  |  |  |
| **CMC 2034** | *C. albicans* | MF767708 | **CMC 1863** | *C. albicans* | MF767794 |  |  |  |
