# Supplementary material for: Merging FT-IR and NGS for simultaneous phenotypic and genotypic identification of pathogenic Candida species
Source: PLoS One. 2017 Dec 4;12(12):e0188104. doi: 10.1371/journal.pone.0188104 (PMC5714347; doi:10.1371/journal.pone.0188104)
Supplement: S3 Table — (DOCX) [file pone.0188104.s003.docx]

| **Strain** | **Type Strain** | **%Identity** | **%Reference Coverage** | **Strain** | **Type Strain** | **%Identity** | **%Reference Coverage** |
| --- | --- | --- | --- | --- | --- | --- | --- |
| **Number** |  |  |  | **Number** |  |  |  |
| **CMC 1965** | *C. albicans* | 99.80 | 95.47 | **CMC 1913** | *C. albicans* | 99.51 | 99.50 |
| **CMC 1966** | *C. albicans* | 99.80 | 95.47 | **CMC 1914** | *C. albicans* | 100 | 99.24 |
| **CMC 1969** | *C. albicans* | 99.80 | 97.71 | **CMC 1915** | *C. albicans* | 99.80 | 98.49 |
| **CMC 1970** | *C. albicans* | 99.71 | 98.81 | **CMC 1918** | *C. albicans* | 99.71 | 98.33 |
| **CMC 1974** | *C. albicans* | 99.61 | 98.31 | **CMC 1919** | *C. albicans* | 99.71 | 99.62 |
| **CMC 1977** | *C. albicans* | 99.71 | 98.01 | **CMC 1920** | *C. albicans* | 99.51 | 99.34 |
| **CMC 1980** | *C. albicans* | 99.80 | 98.87 | **CMC 1921** | *C. albicans* | 99.80 | 99.62 |
| **CMC 1982** | *C. albicans* | 99.80 | 99.16 | **CMC 1923** | *C. albicans* | 100 | 99.61 |
| **CMC 1983** | *C. albicans* | 99.80 | 98.89 | **CMC 1925** | *C. albicans* | 99.51 | 97.30 |
| **CMC 1985** | *C. albicans* | 99.61 | 99.15 | **CMC 1926** | *C. albicans* | 99.90 | 99.18 |
| **CMC 1986** | *C. albicans* | 99.51 | 97.57 | **CMC 1927** | *C. albicans* | 99.61 | 99.71 |
| **CMC 1987** | *C. albicans* | 99.51 | 87.76 | **CMC 1928** | *C. albicans* | 99.71 | 96.91 |
| **CMC 1988** | *C. albicans* | 96.11 | 95.53 | **CMC 1931** | *C. albicans* | 99.51 | 98.34 |
| **CMC 1990** | *C. albicans* | 100 | 98.81 | **CMC 1932** | *C. albicans* | 99.71 | 99.56 |
| **CMC 1991** | *C. albicans* | 99.80 | 97.66 | **CMC 1936** | *C. albicans* | 99.80 | 99.75 |
| **CMC 1992** | *C. albicans* | 99.51 | 98.24 | **CMC 1937** | *C. albicans* | 99.61 | 99.46 |
| **CMC 1994** | *C. albicans* | 99.51 | 96.71 | **CMC 1940** | *C. albicans* | 99.71 | 98.07 |
| **CMC 1998** | *C. albicans* | 99.80 | 99.11 | **CMC 1941** | *C. albicans* | 99.71 | 99.46 |
| **CMC 2000** | *C. albicans* | 99.90 | 96.58 | **CMC 1942** | *C. albicans* | 99.80 | 99.73 |
| **CMC 2001** | *C. albicans* | 99.80 | 99.04 | **CMC 1946** | *C. albicans* | 99.71 | 99.68 |
| **CMC 2008** | *C. albicans* | 99.71 | 97.41 | **CMC 1952** | *C. albicans* | 99.61 | 99.38 |
| **CMC 2019** | *C. albicans* | 99.80 | 98.09 | **CMC 1954** | *C. albicans* | 99.80 | 97.23 |
| **CMC 2020** | *C. albicans* | 99.71 | 97.44 | **CMC 1957** | *C. albicans* | 99.80 | 99.57 |
| **CMC 2023** | *C. albicans* | 99.51 | 98.27 | **CMC 1958** | *C. albicans* | 99.51 | 98.32 |
| **CMC 2025** | *C. albicans* | 99.80 | 98.57 | **CMC 1959** | *C. albicans* | 99.71 | 99.58 |
| **CMC 2026** | *C. albicans* | 99.80 | 97.06 | **CMC 1960** | *C. albicans* | 99.71 | 97.42 |
| **CMC 2029** | *C. albicans* | 99.61 | 97.98 | **CMC 1962** | *C. albicans* | 99.61 | 99.52 |
| **CMC 2030** | *C. albicans* | 99.80 | 97.91 | **CMC 1963** | *C. albicans* | 99.80 | 99.78 |
| **CMC 2031** | *C. albicans* | 99.80 | 98.74 | **CMC 1976** | *C. glabrata* | 99.56 | 98.50 |
| **CMC 2033** | *C. albicans* | 99.71 | 99.06 | **CMC 1989** | *C. glabrata* | 99.64 | 96.15 |
| **CMC 2034** | *C. albicans* | 99.80 | 98.95 | **CMC 2007** | *C. glabrata* | 99.64 | 98.11 |
| **CMC 2035** | *C. albicans* | 99.80 | 98.46 | **CMC 2015** | *C. glabrata* | 99.20 | 88.19 |
| **CMC 2036** | *C. albicans* | 100 | 98.01 | **CMC 2018** | *C. glabrata* | 99.56 | 98.89 |
| **CMC 2037** | *C. albicans* | 99.51 | 98.52 | **CMC 2027** | *C. glabrata* | 99.56 | 98.71 |
| **CMC 2042** | *C. albicans* | 99.61 | 97.70 | **CMC 2032** | *C. glabrata* | 95.09 | 99.06 |
| **CMC 2043** | *C. albicans* | 99.61 | 98.01 | **CMC 1782** | *C. glabrata* | 99.56 | 87.16 |
| **CMC 2045** | *C. albicans* | 99.51 | 98.22 | **CMC 1807** | *C. glabrata* | 99.64 | 98.07 |
| **CMC 2046** | *C. albicans* | 99.80 | 97.37 | **CMC 1813** | *C. glabrata* | 99.56 | 92.82 |
| **CMC 2048** | *C. albicans* | 99.80 | 99.33 | **CMC 1817** | *C. glabrata* | 99.56 | 93.50 |
| **CMC 2049** | *C. albicans* | 99.80 | 99.07 | **CMC 1830** | *C. glabrata* | 99.64 | 93.94 |
| **CMC 2053** | *C. albicans* | 100 | 97.33 | **CMC 1832** | *C. glabrata* | 94.06 | 97.46 |
| **CMC 1768** | *C. albicans* | 99.80 | 97.48 | **CMC 1837** | *C. glabrata* | 99.56 | 98.56 |
| **CMC 1769** | *C. albicans* | 99.80 | 98.36 | **CMC 1846** | *C. glabrata* | 99.27 | 98.08 |
| **CMC 1770** | *C. albicans* | 99.80 | 98.51 | **CMC 1857** | *C. glabrata* | 99.64 | 97.96 |
| **CMC 1771** | *C. albicans* | 99.80 | 98.76 | **CMC 1860** | *C. glabrata* | 95.08 | 99.40 |
| **CMC 1773** | *C. albicans* | 99.80 | 97.82 | **CMC 1861** | *C. glabrata* | 99.49 | 94.27 |
| **CMC 1774** | *C. albicans* | 99.80 | 99.80 | **CMC 1864** | *C. glabrata* | 99.56 | 90.99 |
| **CMC 1776** | *C. albicans* | 99.80 | 99.54 | **CMC 1865** | *C. glabrata* | 99.56 | 92.35 |
| **CMC 1777** | *C. albicans* | 96.11 | 99.49 | **CMC 1884** | *C. glabrata* | 99.27 | 98.99 |
| **CMC 1778** | *C. albicans* | 99.61 | 99.42 | **CMC 1894** | *C. glabrata* | 94.80 | 82.27 |
| **CMC 1780** | *C. albicans* | 99.71 | 99.59 | **CMC 1895** | *C. glabrata* | 99.64 | 92.28 |
| **CMC 1785** | *C. albicans* | 99.51 | 99.62 | **CMC 1912** | *C. glabrata* | 99.27 | 94.02 |
| **CMC 1786** | *C. albicans* | 99.80 | 99.57 | **CMC 1916** | *C. glabrata* | 99.56 | 98.11 |
| **CMC 1788** | *C. albicans* | 99.90 | 99.44 | **CMC 1933** | *C. glabrata* | 99.64 | 92.74 |
| **CMC 1790** | *C. albicans* | 99.51 | 99.47 | **CMC 1934** | *C. glabrata* | 99.56 | 98.84 |
| **CMC 1794** | *C. albicans* | 99.80 | 99.12 | **CMC 1938** | *C. glabrata* | 99.64 | 98.46 |
| **CMC 1795** | *C. albicans* | 99.80 | 99.01 | **CMC 1950** | *C. glabrata* | 99.56 | 99.06 |
| **CMC 1797** | *C. albicans* | 99.80 | 99.19 | **CMC 1964** | *C. glabrata* | 99.56 | 96.26 |
| **CMC 1799** | *C. albicans* | 99.51 | 99.34 | **CMC 1972** | *C. parapsilosis* | 99.36 | 95.77 |
| **CMC 1802** | *C. albicans* | 99.71 | 98.11 | **CMC 1973** | *C. parapsilosis* | 99.54 | 98.95 |
| **CMC 1803** | *C. albicans* | 99.71 | 93.84 | **CMC 1979** | *C. parapsilosis* | 99.54 | 98.75 |
| **CMC 1804** | *C. albicans* | 99.22 | 98.91 | **CMC 1981** | *C. parapsilosis* | 99.54 | 99.01 |
| **CMC 1806** | *C. albicans* | 99.80 | 99.07 | **CMC 2006** | *C. parapsilosis* | 99.54 | 99.04 |
| **CMC 1811** | *C. albicans* | 99.51 | 99.39 | **CMC 2012** | *C. parapsilosis* | 99.54 | 99.00 |
| **CMC 1815** | *C. albicans* | 99.61 | 98.13 | **CMC 2013** | *C. parapsilosis* | 99.54 | 99.28 |
| **CMC 1816** | *C. albicans* | 99.80 | 98.75 | **CMC 2014** | *C. parapsilosis* | 99.54 | 99.12 |
| **CMC 1818** | *C. albicans* | 99.80 | 98.60 | **CMC 2016** | *C. parapsilosis* | 99.54 | 99.07 |
| **CMC 1819** | *C. albicans* | 99.61 | 98.66 | **CMC 2022** | *C. parapsilosis* | 99.54 | 99.02 |
| **CMC 1820** | *C. albicans* | 99.61 | 98.83 | **CMC 2038** | *C. parapsilosis* | 99.45 | 98.67 |
| **CMC 1821** | *C. albicans* | 99.61 | 98.21 | **CMC 2039** | *C. parapsilosis* | 99.54 | 99.09 |
| **CMC 1822** | *C. albicans* | 99.71 | 98.44 | **CMC 2040** | *C. parapsilosis* | 99.45 | 99.23 |
| **CMC 1823** | *C. albicans* | 99.76 | 99.08 | **CMC 2044** | *C. parapsilosis* | 99.54 | 99.24 |
| **CMC 1824** | *C. albicans* | 99.71 | 99.45 | **CMC 2050** | *C. parapsilosis* | 97.18 | 95.36 |
| **CMC 1828** | *C. albicans* | 99.80 | 97.64 | **CMC 1772** | *C. parapsilosis* | 99.45 | 97.30 |
| **CMC 1829** | *C. albicans* | 99.51 | 98.50 | **CMC 1781** | *C. parapsilosis* | 89.68 | 94.61 |
| **CMC 1831** | *C. albicans* | 99.61 | 99.71 | **CMC 1783** | *C. parapsilosis* | 99.45 | 97.93 |
| **CMC 1833** | *C. albicans* | 99.61 | 99.09 | **CMC 1787** | *C. parapsilosis* | 99.54 | 99.27 |
| **CMC 1834** | *C. albicans* | 99.71 | 99.47 | **CMC 1791** | *C. parapsilosis* | 99.54 | 99.13 |
| **CMC 1835** | *C. albicans* | 99.80 | 99.57 | **CMC 1792** | *C. parapsilosis* | 95.96 | 88.86 |
| **CMC 1840** | *C. albicans* | 99.61 | 99.49 | **CMC 1793** | *C. parapsilosis* | 99.54 | 99.03 |
| **CMC 1842** | *C. albicans* | 99.80 | 99.40 | **CMC 1796** | *C. parapsilosis* | 92.53 | 98.04 |
| **CMC 1843** | *C. albicans* | 99.61 | 98.16 | **CMC 1800** | *C. parapsilosis* | 99.27 | 96.58 |
| **CMC 1844** | *C. albicans* | 99.80 | 99.09 | **CMC 1801** | *C. parapsilosis* | 99.50 | 99.25 |
| **CMC 1845** | *C. albicans* | 99.71 | 98.09 | **CMC 1805** | *C. parapsilosis* | 99.54 | 99.11 |
| **CMC 1847** | *C. albicans* | 99.41 | 99.37 | **CMC 1808** | *C. parapsilosis* | 97.08 | 97.67 |
| **CMC 1848** | *C. albicans* | 99.41 | 94.74 | **CMC 1809** | *C. parapsilosis* | 99.45 | 98.98 |
| **CMC 1849** | *C. albicans* | 94.62 | 97.48 | **CMC 1812** | *C. parapsilosis* | 97.10 | 98.31 |
| **CMC 1850** | *C. albicans* | 99.51 | 99.42 | **CMC 1814** | *C. parapsilosis* | 99.45 | 99.00 |
| **CMC 1852** | *C. albicans* | 99.80 | 99.50 | **CMC 1826** | *C. parapsilosis* | 96.82 | 97.98 |
| **CMC 1853** | *C. albicans* | 99.51 | 98.93 | **CMC 1838** | *C. parapsilosis* | 99.63 | 98.07 |
| **CMC 1854** | *C. albicans* | 99.51 | 98.60 | **CMC 1841** | *C. parapsilosis* | 99.54 | 98.66 |
| **CMC 1856** | *C. albicans* | 99.80 | 99.75 | **CMC 1851** | *C. parapsilosis* | 99.63 | 98.46 |
| **CMC 1858** | *C. albicans* | 99.80 | 96.96 | **CMC 1859** | *C. parapsilosis* | 99.63 | 99.20 |
| **CMC 1862** | *C. albicans* | 99.80 | 98.57 | **CMC 1867** | *C. parapsilosis* | 99.45 | 97.52 |
| **CMC 1863** | *C. albicans* | 99.71 | 96.65 | **CMC 1880** | *C. parapsilosis* | 97.08 | 98.39 |
| **CMC 1866** | *C. albicans* | 99.61 | 99.53 | **CMC 1892** | *C. parapsilosis* | 99.63 | 98.56 |
| **CMC 1868** | *C. albicans* | 99.51 | 99.62 | **CMC 1899** | *C. parapsilosis* | 99.59 | 98.67 |
| **CMC 1869** | *C. albicans* | 99.80 | 96.29 | **CMC 1902** | *C. parapsilosis* | 99.45 | 98.23 |
| **CMC 1870** | *C. albicans* | 99.80 | 99.06 | **CMC 1909** | *C. parapsilosis* | 92.78 | 98.49 |
| **CMC 1871** | *C. albicans* | 99.80 | 99.62 | **CMC 1917** | *C. parapsilosis* | 99.63 | 98.77 |
| **CMC 1872** | *C. albicans* | 99.61 | 97.92 | **CMC 1922** | *C. parapsilosis* | 97.26 | 98.97 |
| **CMC 1873** | *C. albicans* | 99.71 | 99.73 | **CMC 1929** | *C. parapsilosis* | 99.54 | 97.09 |
| **CMC 1875** | *C. albicans* | 99.90 | 99.70 | **CMC 1930** | *C. parapsilosis* | 99.54 | 97.54 |
| **CMC 1876** | *C. albicans* | 99.80 | 99.27 | **CMC 1935** | *C. parapsilosis* | 99.45 | 98.57 |
| **CMC 1877** | *C. albicans* | 99.80 | 99.75 | **CMC 1939** | *C. parapsilosis* | 99.54 | 96.92 |
| **CMC 1878** | *C. albicans* | 99.80 | 99.60 | **CMC 1945** | *C. parapsilosis* | 99.63 | 98.83 |
| **CMC 1879** | *C. albicans* | 99.90 | 99.66 | **CMC 1948** | *C. parapsilosis* | 99.63 | 99.30 |
| **CMC 1881** | *C. albicans* | 100 | 99.79 | **CMC 1949** | *C. parapsilosis* | 99.54 | 94.54 |
| **CMC 1885** | *C. albicans* | 99.71 | 99.53 | **CMC 1951** | *C. parapsilosis* | 99.63 | 99.28 |
| **CMC 1886** | *C. albicans* | 99.80 | 99.30 | **CMC 1978** | *C. tropicalis* | 98.47 | 98.38 |
| **CMC 1887** | *C. albicans* | 99.71 | 97.66 | **CMC 2003** | *C. tropicalis* | 98.65 | 97.18 |
| **CMC 1888** | *C. albicans* | 99.80 | 97.63 | **CMC 2009** | *C. tropicalis* | 98.65 | 95.93 |
| **CMC 1889** | *C. albicans* | 99.61 | 99.76 | **CMC 2017** | *C. tropicalis* | 98.47 | 97.10 |
| **CMC 1890** | *C. albicans* | 99.90 | 99.09 | **CMC 2024** | *C. tropicalis* | 98.65 | 98.53 |
| **CMC 1891** | *C. albicans* | 99.80 | 99.48 | **CMC 2041** | *C. tropicalis* | 98.65 | 97.43 |
| **CMC 1893** | *C. albicans* | 99.71 | 85.12 | **CMC 2052** | *C. tropicalis* | 98.56 | 91.94 |
| **CMC 1896** | *C. albicans* | 100 | 99.49 | **CMC 1784** | *C. tropicalis* | 98.53 | 93.50 |
| **CMC 1897** | *C. albicans* | 94.52 | 98.92 | **CMC 1798** | *C. tropicalis* | 98.65 | 95.27 |
| **CMC 1898** | *C. albicans* | 99.51 | 99.47 | **CMC 1810** | *C. tropicalis* | 98.44 | 95.26 |
| **CMC 1900** | *C. albicans* | 99.80 | 99.80 | **CMC 1827** | *C. tropicalis* | 92.83 | 93.30 |
| **CMC 1901** | *C. albicans* | 99.51 | 98.90 | **CMC 1836** | *C. tropicalis* | 98.53 | 94.51 |
| **CMC 1903** | *C. albicans* | 99.71 | 98.66 | **CMC 1839** | *C. tropicalis* | 98.10 | 93.01 |
| **CMC 1905** | *C. albicans* | 99.41 | 99.57 | **CMC 1855** | *C. tropicalis* | 98.56 | 92.97 |
| **CMC 1906** | *C. albicans* | 99.71 | 99.04 | **CMC 1874** | *C. tropicalis* | 98.56 | 96.98 |
| **CMC 1907** | *C. albicans* | 99.90 | 98.75 | **CMC 1904** | *C. tropicalis* | 98.44 | 88.34 |
| **CMC 1908** | *C. albicans* | 96.20 | 99.71 | **CMC 1953** | *C. tropicalis* | 98.47 | 94.98 |
| **CMC 1910** | *C. albicans* | 99.71 | 99.47 | **CMC 1956** | *C. tropicalis* | 98.56 | 97.57 |
| **CMC 1911** | *C. albicans* | 99.71 | 99.52 | **CMC 1961** | *C. tropicalis* | 98.47 | 94.00 |
